# A double blinded, placebo-controlled pilot study to examine reduction of CD34 ^+^/CD117 ^+^/CD133 ^+^ lymphoma progenitor cells and duration of remission induced by neoadjuvant valspodar in dogs with large B-cell lymphoma

**DOI:** 10.12688/f1000research.6055.3

**Published:** 2017-04-18

**Authors:** Daisuke Ito, Michael Childress, Nicola Mason, Amber Winter, Timothy O’Brien, Michael Henson, Antonella Borgatti, Mitzi Lewellen, Erika Krick, Jane Stewart, Sarah Lahrman, Bartek Rajwa, Milcah C Scott, Davis Seelig, Joseph Koopmeiners, Stephan Ruetz, Jaime Modiano

**Affiliations:** 1 Animal Cancer Care and Research Program, College of Veterinary Medicine, University of Minnesota, St. Paul, MN, 55108, USA; 2 Department of Veterinary Clinical Sciences, College of Veterinary Medicine, University of Minnesota, St. Paul, MN, 55108, USA; 3 Masonic Cancer Center, University of Minnesota, Minneapolis, MN, 55455, USA; 4 Department of Veterinary Clinical Sciences, Purdue University College of Veterinary Medicine, West Lafayette, IN, 47907, USA; 5 Department of Clinical Studies, University of Pennsylvania School of Veterinary Medicine, Philadelphia, PA, 19104, USA; 6 Department of Pathology, University of Pennsylvania School of Veterinary Medicine, Philadelphia, PA, 19104, USA; 7 Clinical Investigation Center, College of Veterinary Medicine, University of Minnesota, St. Paul, MN, 55108, USA; 8 Department of Veterinary Population Medicine, College of Veterinary Medicine, University of Minnesota, St. Paul, MN, 55108, USA; 9 Stem Cell Institute, University of Minnesota, Minneapolis, MN, 55455, USA; 10 Department of Basic Medical Sciences, Purdue University College of Veterinary Medicine, West Lafayette, IN, 47907, USA; 11 Division of Biostatistics, School of Public Health, University of Minnesota, Minneapolis, MN, 55455, USA; 12 Novartis Pharma AG, Basel, 4056, Switzerland; 13 Center for Immunology, University of Minnesota, Minneapolis, MN, 55455, USA

**Keywords:** canine, non-Hodgkin, lymphoma, lymphoma, progenitor, cells, ABCB1/P-glycoprotein, valspodar

## Abstract

We previously described a population of lymphoid progenitor cells (LPCs) in canine B-cell lymphoma defined by retention of the early progenitor markers CD34 and CD117 and “slow proliferation” molecular signatures that persist in the xenotransplantation setting. We examined whether valspodar, a selective inhibitor of the ATP binding cassette B1 transporter (ABCB1, a.k.a., p-glycoprotein/multidrug resistance protein-1) used in the neoadjuvant setting would sensitize LPCs to doxorubicin and extend the length of remission in dogs with therapy naïve large B-cell lymphoma. Twenty dogs were enrolled into a double-blinded, placebo controlled study where experimental and control groups received oral valspodar (7.5 mg/kg) or placebo, respectively, twice daily for five days followed by five treatments with doxorubicin 21 days apart with a reduction in the first dose to mitigate the potential side effects of ABCB1 inhibition. Lymph node and blood LPCs were quantified at diagnosis, on the fourth day of neoadjuvant period, and 1-week after the first chemotherapy dose. Valspodar therapy was well tolerated. There were no differences between groups in total LPCs in lymph nodes or peripheral blood, nor in event-free survival or overall survival. Overall, we conclude that valspodar can be administered safely in the neoadjuvant setting for canine B-cell lymphoma; however, its use to attenuate ABCB1 ^+^ cells does not alter the composition of lymph node or blood LPCs, and it does not appear to be sufficient to prolong doxorubicin-dependent remissions in this setting.

## Introduction

The importance of tumor-propagating cells in the pathogenesis of cancer is becoming increasingly well recognized ^1^. However, there are only a few reports supporting the existence of such cells in human lymphoma cell lines or in transgenic lymphoma mouse models ^2– 5^. Our group identified a subset of lymphoid progenitor cells (LPCs) in primary canine B-cell lymphomas that were characterized by co-expression of hematopoietic progenitor antigens CD34, CD117, and CD133, the B-lymphoid lineage marker CD22, and the common leukocyte antigen CD45 ^6^. These LPCs had phenotypic properties consistent with tumor-initiating or tumor-propagating cells (TIC/TPC); they also persisted in the xenotransplantation setting, suggesting that they were relevant to the biology of this disease *in vivo* ^6^. When compared with the bulk of the tumor cells, LPCs showed significantly lower expression of 44 genes across the genome, mapping to cell cycle and transmembrane signaling pathways ^7^. This indicated that LPCs exhibit the characteristic “slow proliferation” seen in normal bone marrow-derived hematopoietic stem cells and in TIC/TPC in other cancers.

One common feature of TIC/TPC in solid tumors is the expression of ATP-binding cassette (ABC) transporter proteins such as ABCB1 (multidrug resistance protein-1 or P-glycoprotein) and ABCG2 (breast cancer resistance protein) ^8^. ABC transporter proteins confer drug resistance by actively transporting drugs from the intracellular space to the extracellular space, thereby preventing the interaction of these drugs with their intracellular targets. In the case of ABCB1, expression has been shown to confer resistance to vinca alkaloids, anthracyclines, taxanes, epipodophyllotoxins, and other drugs ^9, 10^.

Genome-wide gene expression profiling data showed that mRNAs for ABCB1 and ABCG2 were expressed in several types of spontaneous canine lymphomas, including diffuse large B-cell lymphoma (DLBCL) and marginal zone lymphoma (MZL) ^11^. Valspodar (PSC-833) is a selective ABC transporter inhibitor with an acceptable safety profile. Specifically, valspodar had acceptable toxicity when given alone and in combination with cytotoxic chemotherapy in Phase I/II clinical trials in humans with several types of cancer and in one study of dogs with naturally occurring osteosarcoma treated with doxorubicin ^12– 16^. These favorable toxicological and pharmacokinetic profiles made valspodar an attractive candidate for targeting LPCs, especially because a safe protocol had been previously established for its neoadjuvant use to inhibit ABCB1 in dogs receiving doxorubicin chemotherapy ^14^.

Three large, double blinded, randomized phase-3 clinical trials have been reported using valspodar in combination with chemotherapy to treat people with cancer. One targeted patients with relapsed/refractory multiple myeloma ^17^, another was done in women with stage IV or suboptimally debulked stage III ovarian or primary peritoneal cancer ^18^, and the third enrolled newly diagnosed patients under 60 years old with acute myeloid leukemia ^19^. The addition of valspodar did not improve objective results in any of these studies, and it generally enhanced toxicity associated with chemotherapy. However, none of the studies evaluated whether valspodar sensitized specific cell subpopulations within the tumors, including TICs or TPCs, to the cytotoxic effects of chemotherapy.

We designed this study specifically to test that question; that is, whether neoadjuvant valspodar treatment would enhance the sensitivity of LPCs to doxorubicin in dogs with spontaneous large B-cell lymphomas.

## Materials and methods

### Supplies and reagents

Clinical grade valspodar (PSC-833) was provided by Novartis Pharma AG (Basel, Switzerland). Valspodar was compounded for use in pet dogs by Custom Rx Compounding Pharmacy (Roy D. Katz R. Ph., Richfield, MN). Capsules containing 100 mg valspodar or placebo (compounding materials without valspodar) were formulated with the same method used to compound cyclosporine-A for oral use in dogs, since these compounds share a high degree of structural similarity. Activity of the compounded valspodar was confirmed using the side population assays described below. Research grade valspodar and verapamil were purchased from Sigma-Aldrich (St. Louis, MO) and were diluted in dimethyl sulfoxide (DMSO; Sigma-Aldrich) for use *in vitro*. Lymphoma cells were maintained in short-term culture as described ^6, 20, 21^. COSB hemangiosarcoma cells were maintained as adherent cultures as described ^22^.

### Trial design

This was a double blinded, placebo-controlled trial with 10 dogs in each study arm. The main statistical endpoint was a change in LPCs following treatment. The hypothesis was that a significant reduction in the number of LPCs in blood and/or in lymph node cells would occur in dogs treated with valspodar, but not in dogs receiving placebo. The sample size of 10 dogs per group was selected to provide 80% power to establish a difference of ± 2 S.D. in LPCs pre-and post-valspodar or placebo treatment within and between groups. The study was not powered to detect significant differences in duration of remission or overall survival. However, outcomes were recorded to evaluate trends that could be used to design future studies or could be included in meta-analysis. Criteria for inclusion were *(1)* clinical diagnosis of multicentric lymphoma (WHO stage I-V); *(2)* confirmed WHO classification of large B-cell lymphoma (DLBCL or MZL in transition) ^23^; *(3)* favorable performance status with an expected survival time of ≥ 30 days; *(4)* body weight more than 15 kg (to allow adequate blood sampling) and less than 40 kg (to ensure dosing feasibility); *(5)* platelet count ≥100,000/ml and packed cell volume ≥30%; and *(6)* informed pet owner consent in writing. Criteria for exclusion were *(1)* disease substage b; *(2)* any previous therapy for lymphoma, including corticosteroids; *(3)* lymphomas classified as other than DLBCL or MZL in transition; *(4)* dogs from herding breeds with high frequency of inactivating MDR-1 polymorphisms ^24, 25^; and *(5)* significant co-morbidities, such as renal or hepatic failure, congestive heart failure, or clinical coagulopathy. There were no restrictions based on age, gender, neuter status, or other physical parameters.

**Figure 1.**
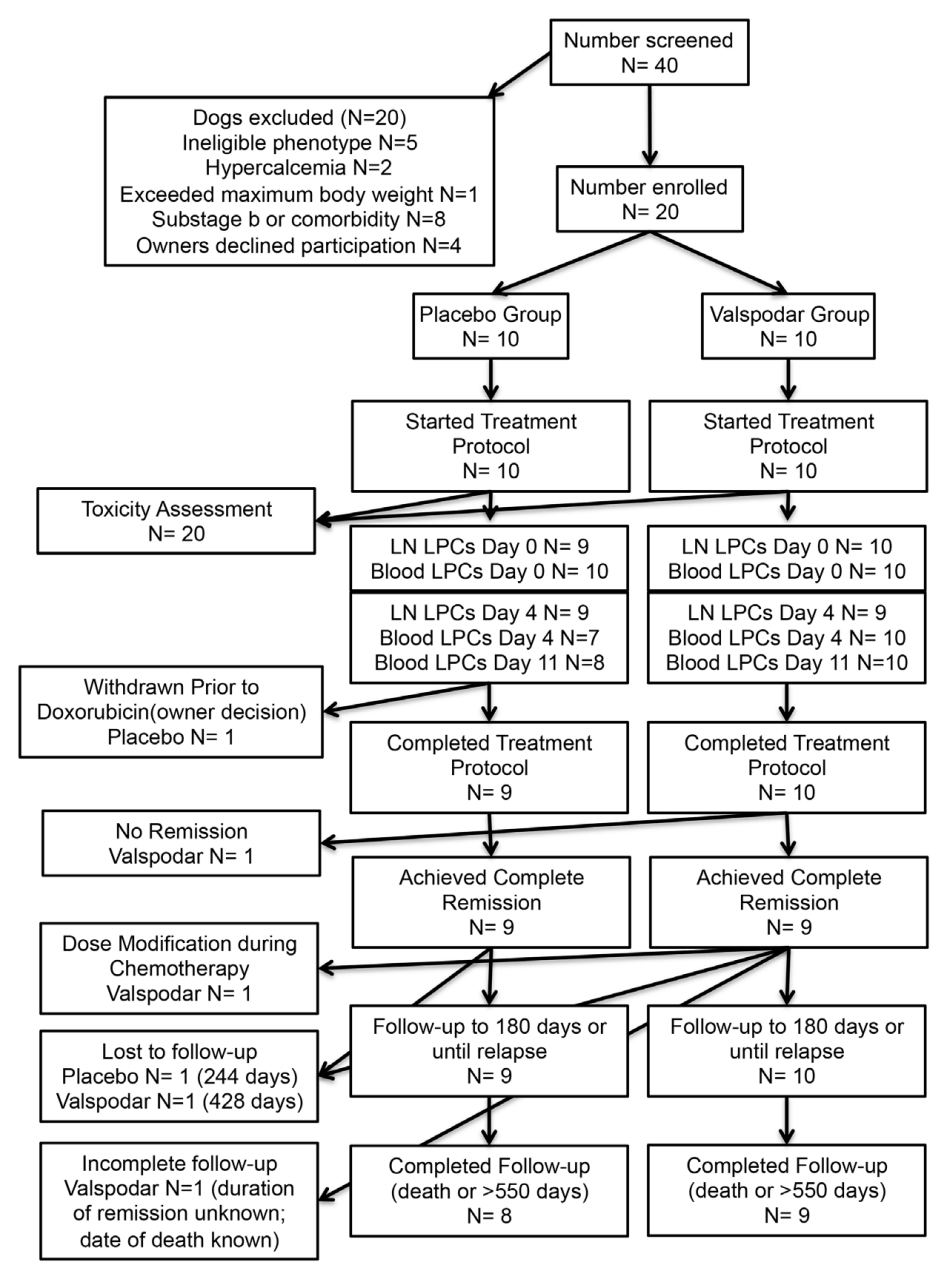
Enrollment, exclusions, and assessments. Flow chart with details of dogs enrolled in the study and exclusions from each of the measured endpoints.

Treatment costs for eligible participants up to $2500 were paid by study funds through the end of the chemotherapy protocol. The study was conducted with approval and under the oversight of the University of Minnesota Institutional Animal Care and Use Committee (IACUC Protocol 1011A92815 “Ablation of tumor initiating cells by P-glycoprotein inhibition: Proof of principle study in canine diffuse large B-cell lymphoma”). The trial design and implementation conformed to the Standard Protocol Items: Recommendations for Interventional Trials (SPIRIT) guidelines ^26^ where they apply to studies in companion animals. The flow of participants is provided in Figure 1. The demographic composition of the study population after unblinding is provided in Table 1. The timing of each procedure is shown in Table 2.

**Table 1.** Signalment (demographic characteristics) of study dogs.

	All Dogs	Placebo Group	Valspodar Group
	20	10	10
Gender Male (neutered) Female (spayed) Not reported	9 (7) 10 (10) 1	4 (3) 5 (5) 1	5 (4) 5 (5) 0
Breed Golden retriever Labrador retriever Vizsla Other (beagle, mixed breed, Springer spaniel, poodle, bulldog) Not reported	6 3 3 7 1	1 1 2 5 1	5 2 1 2 0
Age Median (yr) Mean (yr) Range (yr)	7.6 7.5 3.8 - 12.1	8.4 8.6 5.3 - 12.1	6.8 6.7 3.8 - 11.6
Stage IIIa IVa Va Not reported	6 10 3 1	3 5 1 1	3 5 2 0

**Table 2.** Study protocol.

	Day 0	Day 1	Day 2	Day 3	Day 4	Day 5	Day 11	4X 21 days cycle
Lymph node biopsy	X				X			
Blood and serum samples	X				X		X	
Cytology and histopathology	X				X			
Valspodar/Placebo (7.5 mg/kg, PO, q 12 hr)		X	X	X	X	X		
Doxorubicin (21 mg/m ^2^, IV)					X			
Doxorubicin (30 mg/m ^2^, IV)								X

### Sample handling and pathological classification

Incisional wedge biopsies collected during eligibility screening before treatment (Day 0) and tru-cut biopsies collected on the fourth day of neoadjuvant treatment for enrolled dogs (Day 4) were processed as described ^27^. Briefly, representative sections from each biopsy were fixed in 10% neutral buffered formalin for 24 hours and embedded in paraffin for routine histological analysis. Sample processing, staining, and immunohistochemical stains were done by the Comparative Pathology Shared Resource of the Masonic Cancer Center, University of Minnesota. Samples were classified according to the modified WHO scheme for canine lymphoma based on cell morphology, immunophenotyping using antibodies against human CD3 (AbD Serotec Cat# MCA1477T RRID:AB_10845948), human CD20 (Lab Vision Cat# RB-9013-P0 RRID:AB_149766), and CD79a (clone HM47/A9, Cat# CM 067 C, now supplied by Thermo Scientific as RRID:AB_10981393), and available clinical history by two board certified veterinary pathologists (TDO and DMS). The remainder of the biopsy samples was used to prepare single cell suspensions to support the diagnoses through flow cytometry; these suspensions were cryopreserved in liquid nitrogen storage for the following analyses as described ^6, 27^.

Blood samples were collected in evacuated EDTA tubes at Day 0, Day 4, and Day 11 to monitor toxicity and to evaluate blood LPCs. Adverse events were recorded and classified according to the Veterinary Cooperative Oncology Group (VCOG) criteria ^28^.

### Flow cytometry

*Tumor tissues *- Flow cytometry analysis was performed as described ^6, 20^. Briefly, 5 × 10 ^5 ^ tumor cells were incubated with dog immunoglobulin G (IgG; Jackson ImmunoResearch, West Grove, PA) to prevent non-specific binding of antibodies to Fc receptors. Cells were stained using fluorescein isothiocyanate (FITC), phycoerythrin (PE), or allophycocyanin (APC) and conjugated antibodies against dog CD3 (clone CA17.2A12, AbD Serotec Cat# MCA1774F RRID:AB_2291174), dog CD4 (clone YKIX302.9, AbD Serotec Cat# MCA1038F RRID:AB_321271), dog CD5 (clone YKIX322.3, AbD Serotec Cat# MCA1037F RRID:AB_322643), dog CD8 (clone YCATE55.9, AbD Serotec Cat# MCA1039PE RRID:AB_322646), dog CD45 (clone YKIX716.13, AbD Serotec Cat# MCA1042F RRID:AB_324047, Cat# MCA1042PE RRID:AB_322644, and AbD Serotec Cat# MCA1042APC RRID:AB_324810), dog CD21 (clone CA2.1D6, AbD Serotec Cat# MCA1781PE RRID:AB_323238), human ABCB1 (clone UIC2, eBioscience Cat# 17-2439-42 RRID:AB_10736477), and human ABCG2 (clone 5D3, eBioscience Cat# 12-8888-82 RRID:AB_466219). Anti-human CD22 antibody (clone RFB4, Abcam Cat# ab23620 RRID:AB_447570) was labeled using the Zenon anti-mouse IgG1 Alexa-Fluor 647 labeling kit (Invitrogen-Molecular Probes, Carlsbad, CA). LPCs were detected by a cocktail of antibodies directed against canine CD34 (clone 1H6, BD Biosciences Cat# 559369 RRID:AB_397238), human CD117 (clone YB5.B8, BD Biosciences Cat# 555714 RRID:AB_396058), and mouse CD133 (clone 13A4, eBioscience Cat# 12-1331-80 RRID:AB_465848), where the mix was designated as “Progenitor” ^6^. The antibodies directed against human and mouse antigens have been shown to recognize the canine homologs ^6, 21, 29^. Cells were gated based on their light scatter properties, and dead cells were excluded using *7*-amino-actinomycin D (7-AAD; eBioscience) staining. Flow cytometry was performed using an LSRII cytometer (BD Immunocytometry Systems, San Jose, CA), and results were analyzed using FlowJo software (Tree Star, RRID:nif-0000-30575).

*Peripheral blood *– All flow cytometric experiments on peripheral blood were performed at the Flow Cytometry and Cell Separation Facility within the Bindley Bioscience Center at Purdue University using an iCyt Reflection HAPS cell sorter (Sony Biotechnology, Inc., San Jose, CA). Results were analyzed using FlowJo software (Tree Star, RRID:nif-0000-30575). Data to derive the frequency of LPCs in blood were also analyzed using FCS Express ( *De Novo* Software, Los Angeles, CA). Briefly, approximately 50 ml peripheral blood was collected via jugular venipuncture into EDTA tubes from each study dog on Days 1, 4, and 11. Blood samples collected at the University of Minnesota and the University of Pennsylvania were mixed in a 1:1 ratio with RPMI-1640 (Mediatech, Inc., Manassas, VA) and shipped on ice to Purdue University for flow cytometric analysis. Samples collected at Purdue University were processed immediately for analysis. All blood samples were centrifuged at 1500 × g for 20 minutes at 4°C. Plasma was removed by vacuum suction, and the buffy coat was manually harvested from each sample, then transferred to microcentrifuge tubes. Buffy coats were re-centrifuged at 1500 × g for 15 minutes at 4°C, then re-harvested. Cells were stained using FITC, PE, or APC-conjugated antibodies against human CD22 (clone RFB4, Abcam Cat# ab23620 RRID:AB_447570), canine CD34 (clone 1H6, BD Biosciences Cat# 559369 RRID:AB_397238), human CD117 (clone YB5.B8, BD Biosciences Cat# 555714 RRID:AB_396058), and mouse CD133 (clone 13A4, eBioscience Cat# 12-1331-80 RRID:AB_465848). Isotype control antibodies (mouse IgG1 (eBioscience Cat#12-4714-82) and rat IgG2b (eBioscience Cat#11-4031-81) conjugated to APC were used to exclude dead or irrelevant cells, while LPCs were detected by dual staining with FITC-CD22 and PE-“Progenitor mix” (CD34, CD117, CD133). Assuming that circulating LPCs would be very rare in the peripheral blood, approximately 10 ^8^ cells were sorted at each sampling time point for each dog to provide a reasonable likelihood of identifying this population.

### Side population assays

Side populations were measured as described ^30^. Briefly, DyeCycle Violet (DCV) (Life Technologies, Eugene, OR) was added to a final concentration of 10 μM, and 5 × 10 ^5^ cells were incubated for an additional 60 minutes at 37°C with intermittent mixing. Cells were washed, filtered, and maintained on ice until analysis. To exclude dead cells from analysis, 7-AAD was added to each sample immediately before collection. DCV emission was detected using a BD LSRII flow cytometer (BD Biosciences). Valspodar and verapamil were diluted in DMSO for use in this assay. Equivalent amounts of DMSO were added to control samples, and verapamil was used to determine the side population gates. Data were analyzed using FlowJo software (Tree Star, RRID:nif-0000-30575).

### RNA preparation and RNA sequencing

RNA prepared from biopsies obtained at diagnosis (Day 0) and on the fourth day of neoadjuvant treatment for enrolled dogs (Day 4) was quantified and assessed for quality as described ^11, 22^. Briefly, total RNA was quantified using a fluorimetric RiboGreen assay and the total RNA integrity was assessed using capillary electrophoresis in the Agilent BioAnalyzer 2100 to generate RNA Integrity Numbers (RIN). Samples passed a QC step if they contained >1 µg with a RIN >8. Next-generation RNA sequencing (RNAseq, 50-bp paired-end) with HiSeq 2000 (Illumina) was done at the University of Minnesota Genomics Center (UMGC) in 14-paired (pre- and post-treatment) samples and two additional pre-treatment samples as described ^22^. A minimum of ten million read-pairs was generated for each sample. Illumina’s CASAVA software 1.8.2 was used for verifying the quality of the sequence data and for FASTQ file generation and de-multiplexing. FASTQ files and processed data files are available through the National Center for Bioinformatics (GSE93516).

### Bioinformatic analyses

Initial quality control analysis of the FASTQ files was performed using the FastQC software (v0.11.5; http://www.bioinformatics.babraham.ac.uk/projects/fastqc). Sequenced reads were trimmed for adaptor sequence, and masked for low-complexity or low-quality sequence using Trimmomatic (v0.33) enabled with the optional "- - quality control" option set as: 3bp sliding-window trimming from 3’ end requiring minimum Q16 ^31^. Paired-end sequences were mapped to the CanFam3 reference genome with the HISAT2 aligner (2.0.2-beta) using default parameters ^32^. Metrics for insert size distribution of each paired-end library were calculated using Picard software (version 1.126) ( http://picard.sourceforge.net.). Samtools (v1.2 using htslib-1.2.1) was used to sort and index each bam file ^33^. For each sample, a transcript abundance estimation file was generated with Cuffquant (Cufflinks (v2.2.1)) using default settings and the “–multi-read-correct” option enabled ^34^. Cuffnorm was used to generate a table of fragments per kilobase of exon per million fragments mapped (FPKM) expression data using the classic-fpkm normalization method and the “-p/–num-threads” option set to 16. Cuffdiff with default parameters was used to test differences in the summed FPKM of transcripts sharing each gene_id to get gene level differential expression between two groups ( *i.e.*, pre- and post-treatment). Expression differences with a false discovery rate (FDR)- adjusted p-value (q-value) of less than 0.05 were considered significant. Principal Component Analysis (PCA) was performed on FPKM values with the built-in R function *prcomp* and these results were visualized with the ‘ggplot2’ and ‘ggfortify’ packages in Rstudio (Version 0.99.491).

### Treatment

Eligible dogs were randomized into an experimental treatment group that was given encapsulated valspodar (7.5 mg/kg orally every 12 hours for 5 days) or a control group that was given the equivalent encapsulated placebo over the same schedule. Starting on Day 4, every dog received five doses of doxorubicin 21 days apart using a dosing schedule based on a previous study using valspodar in the neoadjuvant setting with single agent doxorubicin chemotherapy in dogs with osteosarcoma ^14^. The first dose was reduced by 30% from the standard (from 30 mg/m ^2^ to 21 mg/m ^2^) to mitigate potential side effects of ABCB1 inhibition by the neoadjuvant valspodar. If no serious toxic effects of combined doxorubicin/valspodar were observed, subsequent doxorubicin treatments were dosed at 30 mg/m ^2^. If toxic effects were observed, the dose remained at 21 mg/m ^2^ and subsequent dose escalation to 30 mg/m ^2^ only occurred if no serious adverse events were recorded following the previous dose. An overview of the treatment and collection of blood and tissue samples is provided in Table 2. The treatment responses were evaluated based on the VCOG criteria for lymphoma in dogs ^35^. The last treatment was given at 111 days; dogs were examined once more at 180 days, which was near the expected median survival for single agent doxorubicin protocol ^36^, and then released to their attending veterinarian. The status for each dog was ascertained by telephone or electronic mail communication with the attending veterinarians and/or the owners periodically thereafter until a death event was recorded or >500 days had elapsed. Relapse was determined using clinical parameters (generalized lymphadenopathy on physical exam) with conventional testing as needed (routine radiographs or ultrasound imaging, fine needle aspirate). Dogs were considered off-study at relapse and were then eligible to undergo rescue therapy (N=11) or enter other clinical studies (N=4).

### Measurement of valspodar concentration in canine serum

Serum samples collected on the fourth day of neoadjuvant treatment (Day 4) were stored at -80°C until analysis. Valspodar was quantified by liquid chromatography/mass spectroscopy (LC-MS/MS) using a high-performance liquid chromatograph (Agilent 1200 Series, Santa Clara CA) coupled with a TSQ Quantum triple stage quadrupole mass spectrometer (Thermo-Electron, San Jose, CA) as described ^37^.

### Statistics

Descriptive statistics (mean, median, minimum, maximum) were recorded for age, gender, breed, and disease stage; for each variable, differences between groups were determined using Fisher’s exact test. Time to remission, duration of remission, and overall survival were recorded in days starting on the date that the dogs first received a clinical diagnosis. The percentage of LPCs in lymph node samples was calculated based on expression of relevant cell surface markers (CD34/CD117/CD133) as a proportion of live, large, CD22 ^+^ B cells ^6^. The ΔLPC was calculated as the ratio of LPCs at Day 4 over LPCs at Day 0. The Mann-Whitney Test (Prism 5, GraphPad Software, Inc., La Jolla, CA) was used to determine significance between LPC numbers in lymph nodes from dogs in the experimental treatment and in the control groups. The associations between variables were determined using the Pearson correlation. Differences between groups in duration of remission and overall survival were determined using Kaplan-Meier probability and log-rank tests. The proportion of LPCs in peripheral blood was calculated by conservatively gating on CD22/“Progenitor mix” double positive cells identified in two-dimensional scatter plots. However, the very low numbers of LPCs detected in peripheral blood and the presence of random effects (significant intra- and inter-dog variation in light scatter parameters characterizing LPC cells shape and size) made a direct comparison of the identified proportions between dogs challenging. The effect of the therapy on the percentage of cells was expressed therefore as a summary of Cohen’s *h* values computed for every dog, and for every pair of time points. The summary *h* and accompanying 95% confidence intervals were calculated assuming a random-effects model.

## Results

### Inhibition of drug efflux by valspodar *in vitro*

**Figure 2.**
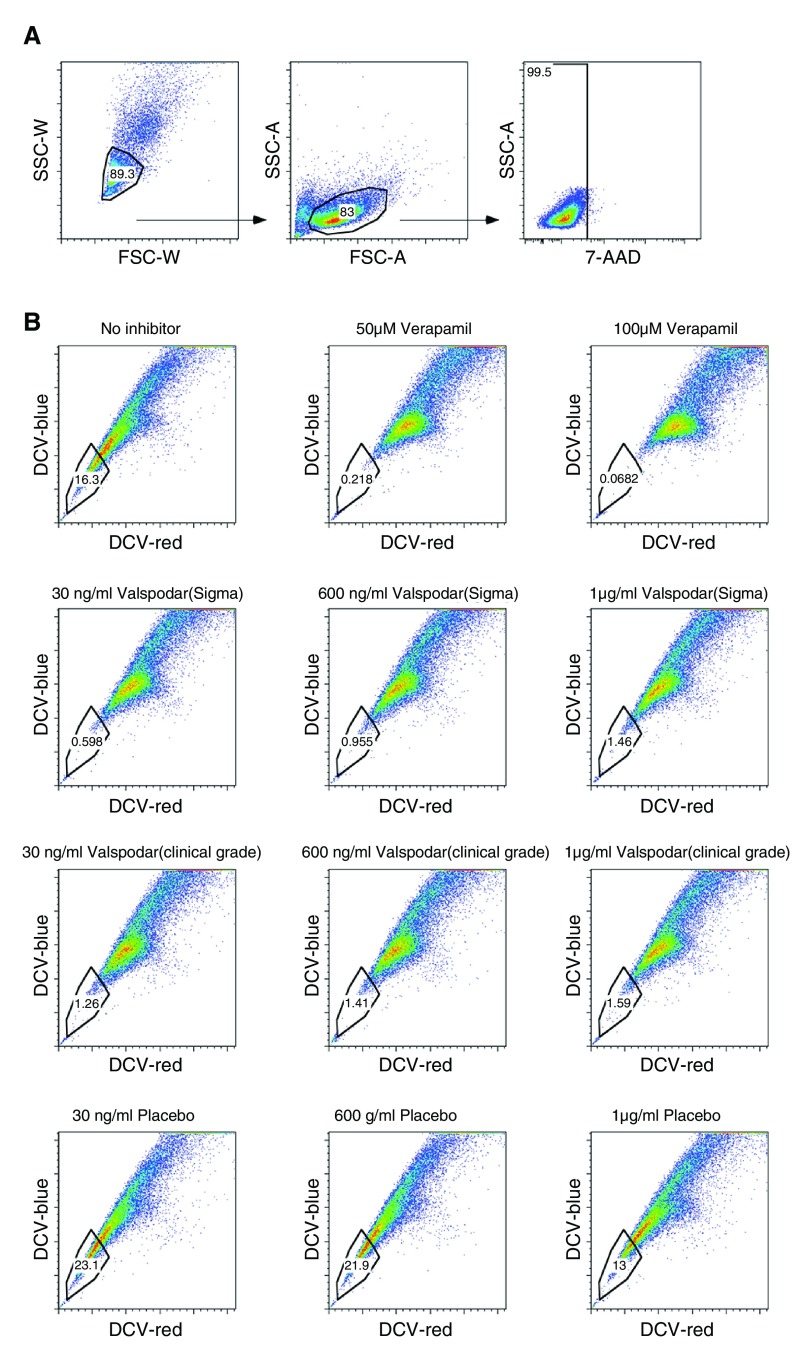
Valspodar inhibits dye excluding side population in canine hemangiosarcoma cells at clinically achievable concentrations. Side population analyses were done as described in Materials and methods using cultured COSB canine hemangiosarcoma cells. ( **A**) Live cells were gated based on light scatter properties and exclusion of 7-AAD, and ( **B**) the side populations were determined based on DyeCycle Violet (DCV) efflux. Verapamil was used to inhibit ABCB1 and ABCG2 at 50–100 µM concentrations. Clinical grade and research grade valspodar was used at concentrations that were achieved in the plasma of dogs in the study (30 – 600 ng/ml) as well as at the saturating dose of 1 µg/ml. The Y-axis is DCV-blue (450+/-50 nm) emission while the X-axis is DCV-red (660 +/- 40 nm) on the LSR-II. Data were analyzed and dot plots were created in FlowJo.

Valspodar is a potent, selective inhibitor of the ABCB1 efflux transporter ^12, 13^. To confirm that the clinical grade compound retained potency after compounding, we examined its effect to inhibit DCV efflux using the flow cytometric side population assay. COSB canine hemangiosarcoma cells contain a subpopulation of cells that shows robust dye efflux in this assay ^30^ ( Figure 2, Dataset 1). The compounded, clinical grade valspodar was as effective as the research grade valspodar in this assay, eliminating >90% of the side population ( *i.e.*, it inhibited dye efflux) at concentrations as low as 30 ng/ml ( Figure 2, Dataset 1). The effect of valspodar was comparable to that observed in verapamil ( Figure 2, Dataset 1), which inhibits both ABCB1 and ABCG2 at the 50–100 µM concentrations used in this assay.

### Recruitment and randomization

Excluding dogs that had received previous chemotherapy, 40 dogs were screened for eligibility. Twenty dogs were eligible and enrolled in the trial. Of the 20 dogs that were excluded, 5 dogs had lymphomas that were classified as other than DLBCL or MZL in transition (specifically, three had T-cell lymphoma, one had an indolent type of lymphoma, and one had disease largely confined to spleen with minimal peripheral lymphadenopathy that precluded biopsy) and 15 dogs had hypercalcemia (N=2), lymphoma in substage b or a ongoing co-morbidity (N=8), exceeded the maximum allowable body weight (N=1), or the owners declined participation (N=4).

Of the twenty dogs enrolled, 10 were randomized to each group. The distribution of dogs according to demographic characteristics is shown in Table 1. The composition of the study population was predictable ^38, 39^, and there were no statistically significant differences in any category between the experimental treatment group and the control group. One dog in the placebo group did not receive doxorubicin chemotherapy after the neoadjuvant period per its owner’s decision. This dog was censored in the outcome assessments.

### Toxicity attributable to valspodar

Six dogs, including three in the placebo group and three in the experimental (valspodar) group, had reportable events during the study ( Table 3). The most common toxicities observed in both groups were grade-1 and grade-2 inappetence, lethargy, vomiting, and diarrhea. No grade-4 or grade-5 toxicities were observed, although one event was potentially dose limiting. One dog had grade-2 hematological toxicity (neutropenia and thrombocytopenia) after the first administration of doxorubicin. The doxorubicin dose for the second administration was maintained at 21 mg/m ^2^ and no toxicity was observed. However, the owner only permitted subsequent doxorubicin doses to be escalated to 24 mg/m ^2^. The dog that was withdrawn after neoadjuvant placebo had grade-2 gastrointestinal toxicity and grade-1 lethargy.

**Table 3.** Reportable events and treatment adjustments.

Dog ID Time of event	Placebo Group	Valspodar Group
MN06 Day 3		Inappetence (grade 1)
MN08 Day 2	Inappetence (grade 2) Lethargy (grade 2)	
PD02 Day 1 ^1^	Gastrointestinal ^2^ (grade 2) Lethargy (grade 1)	
PD05 Day 1 after first dose of doxorubicin ^3^		Lethargy (grade 1) Gastrointestinal ^4^ (grade 2) Hematological ^5^ (grade 2)
PENN02 Day 2 and Day 5 Days 6–11 after first dose of doxorubicin		Gastrointestinal ^6^ (grade 1) Inappetence (grade 3) Lethargy (grade 2)
PENN05 Day 4 Day 11	Bilateral scleral congestion Suspected hyphema OS (grade 2) Lymphadenopathy Uveitis OD (grade 2)	

^1^Owner elected to withdraw dog from study prior to receiving doxorubicin ^2^Vomiting and diarrhea ^3^Dog’s second doxorubicin treatment was dosed at 21 mg/m ^2^; similar toxic effects were not observed. However, the dog’s owner only permitted subsequent doxorubicin doses to be escalated to 24 mg/m ^2^ ^4^Diarrhea ^5^Neutropenia (grade 2) and thrombocytopenia (grade 1) ^6^Vomiting

### Quantification of LPCs in blood and lymph nodes from dogs with lymphoma

**Figure 3.**
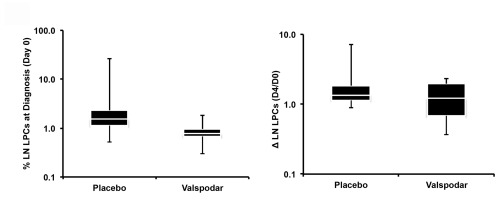
LPCs in lymph nodes from dogs with large B-cell lymphoma at diagnosis and on the fourth day of neoadjuvant treatment with valspodar. *Left*: Box plots showing median (white line), 75% confidence intervals, and outliers of the percent LPCs in lymph nodes at diagnosis, and *Right*: relative change in LPCs from the time of diagnosis (Day 0) to the fourth day of the neoadjuvant period (Day 4) in each group of dogs. A ΔLPC = 1.0 means no change in the percent LPCs measured at both time points. Data were analyzed and graphs were assembled using MS Excel.

**Figure 4.**
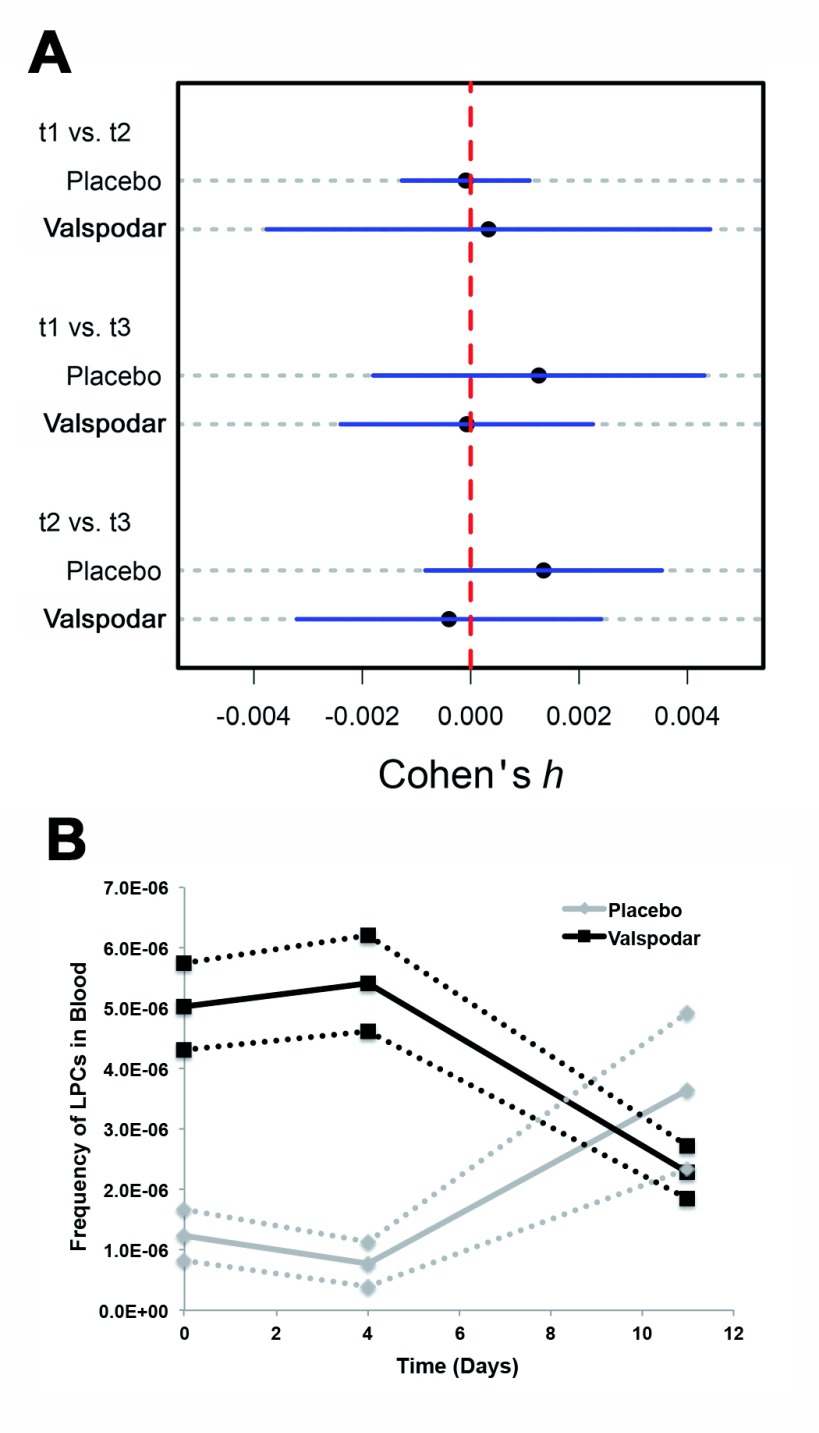
Effect of valspodar treatment on depletion of peripheral blood LPCs in dogs with diffuse large B-cell lymphoma. ( **A**) Plots of effect sizes (Cohen’s *h*), with corresponding 95% confidence intervals, associated with valspodar treatment on LPC frequency in peripheral blood. Effect sizes of valspodar treatment on changes in LPC frequency between all sampling time points are depicted. All calculated effect sizes were small, suggesting no effect of therapy on changes in peripheral blood LPC proportion between time points. However, the 95% confidence intervals are wide due to high variability in sample quality. t1=Day 0; t2=Day 4; t3=Day 11. ( **B**) Mean frequency of blood LPCs from Day 0 to Day 11 in dogs receiving placebo (red lines, squares) and in dogs receiving valspodar (green lines, triangles). Dotted lines denote the 95% lower and upper boundaries of LPC frequencies for each group.

Blood and lymph node LPCs were quantified for each dog at diagnosis (Day 0) and on the fourth day of neoadjuvant treatment (Day 4) as described in Materials and Methods. Blood LPCs were also quantified for each dog 7 days after doxorubicin treatment (Day 11). The distribution of lymph node LPCs at diagnosis was narrower in the dogs that received valspodar than in the control dogs ( Figure 3A), but the two groups were not significantly different, and neither group showed a statistically significant reduction in LPCs on the fourth day of the neoadjuvant period (ΔLPC). Table 4 shows that LPCs were detectable in every peripheral blood sample; however, at a frequency lower than what was previously reported in lymph nodes ^6^. The effects of treatment on peripheral blood LPCs, as described by Cohen’s *h*, were extremely small and characterized by wide confidence intervals ( Figure 4A). There was an intriguing reversal of trends in the frequency of blood LPCs in dogs treated with valspodar and dogs treated with placebo at day 11 (one week after administration of doxorubicin, Figure 4B). However, this must be interpreted cautiously. First, we must note that the number of blood LPCs were not significantly different (p=0.18) using analysis of variance (ANOVA). Second, large variances were present within each group, as showed by the Cohen’s *h* ( Figure 4A). And finally, the number of LPCs in all the samples remained within the relatively wide range observed in pre-treatment samples. The low abundance of LPC cells means that even a decrease in frequency by an order of magnitude would result in an effect size no larger than *h*=0.0013 per dog. Representative dot plots illustrating gating and presence of LPCs in peripheral blood are depicted in Supplemental Figures 1A – 1D.

**Table 4.** Changes in proportion of LPCs in peripheral blood ^1^.

	Ratio	95% LB	95% UB
	Placebo		
**t _1_**	1 : 8.040 × 10 ^5^	1 : 1.225 × 10 ^6^	1 : 5.983 × 10 ^5^
**t _2_**	1 : 1.314 × 10 ^6^	1 : 2.537 × 10 ^6^	1 : 8.865 × 10 ^5^
**t _3_**	1 : 2.751 × 10 ^5^	1 : 4.274 × 10 ^5^	1 : 2.028 × 10 ^5^
	**Valspodar**		
**t _1_**	1 : 1.989 × 10 ^5^	1 : 2.326 × 10 ^5^	1 : 1.737 × 10 ^5^
**t _2_**	1 : 1.848 × 10 ^5^	1 : 2.168 × 10 ^5^	1 : 1.611 × 10 ^5^
**t _3_**	1 : 4.377 × 10 ^5^	1 : 5.417 × 10 ^5^	1 : 3.672 × 10 ^5^

^1^ Frequency of lymphoid progenitor cells (LPCs) in peripheral blood, expressed as a ratio of LPCs over all peripheral blood leukocytes. The values reported in the first column represent mean proportions of LPCs detected across all study dogs at each time point. The second and third columns represent the lower (95% LB) and upper (95% UB) boundaries of the 95% confidence intervals for the values in the first column. t _1_ = Day 0, t _2_ = Day 4, t _3_ = Day 11.

### Alterations in ABCB1 and ABCG2 expression by LPCs in lymph nodes from dogs with lymphoma

**Figure 5.**
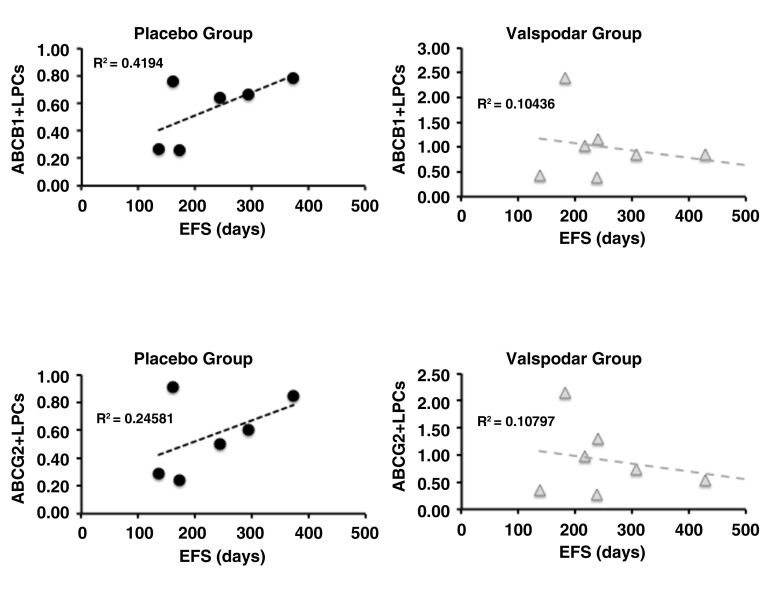
Event-free survival of dogs as a function of ABCB1 and ABCG2 expression in lymph node LPCs from dogs with large B-cell lymphoma at diagnosis. Dot plots showing the relationship between ABCB1 expression and event-free survival (EFS) in days (top) and between ABCG2 expression and EFS in days (bottom) in dogs treated with placebo (N = 9) or with neoadjuvant valspodar (N = 9) where samples were available for these measurements. The dashed lines represent linear regressions and their R ^2^ values are indicated on each graph. The Y-axis represents the % of ABC ^+^/Progenitor ^+^lymph node B cells. Data were analyzed and graphs were assembled using MS Excel.

The absence of a treatment effect on total LPCs suggested that we should not reject the null hypothesis that neoadjuvant valspodar did not enhance chemosensitivity of LPCs, and could reflect variable expression of ABC transporters by these cells. Samples from 15 dogs in the study (six in the placebo group and nine in the valspodar group) had sufficient material for analysis of ABCB1 and ABCG2 expression in LPCs at diagnosis. The proportion of ABCB1 ^+^ LPCs and ABCG2 ^+^ LPCs was variable. In the placebo group, between 1.6% and 52.4% of lymph node LPCs expressed these proteins at the time of diagnosis; in the valspodar group, the range of ABCB1 and ABCG2 transporter expression in lymph node LPCs at the time of diagnosis was 10.0% to 72.7% ( Table 5). When we examined the proportion of ABCB1 ^+^ LPCs and ABCG2 ^+^ LPCs in dogs from each treatment group, we saw an intriguing reversal in the trends with regard to event-free survival ( Figure 5), although neither group showed a significant correlation between the number of ABCB1 ^+^ or ABCG2 ^+^ cells at diagnosis and survival (all the R ^2^ values were less than or equal to 0.42).

**Table 5A.** Frequency of ABCB1 ^+^ and ABCG2 ^+^ LPCs in lymph nodes.

	All Dogs (N=15)	Placebo Group (N=6)	Valspodar Group (N=9)
% ABCB1+ LPCs (Mean) (Median) (Range)	33.4 34.3 1.7 - 72.7	20.2 15.2 1.7 - 43.4	42.3 45.3 10 - 72.7
% ABCG2+ LPCs (Mean) (Median) (Range)	32.3 35.7 1.6 - 68.8	22.2 15.2 1.6 - 52.4	39.0 38.9 10.9 - 68.8

**Table 5B.** Valspodar-induced alterations in ABCB1 ^+^ and ABCG2 ^+^ LPCs in lymph node.

Dog ID	Group	% ABCB1 ^+^ LPCs	% ABCG2 ^+^ LPCs
MN05 Day 0 Day 4	Placebo	43.4 36.6	52.4 22.1
MN09 Day 0 Day 4	Placebo	1.7 1.1	1.6 0.8
MN02 Day 0 Day 4	Valspodar	60.8 47.9	68.8 61.3
MN10 Day 0 Day 4	Valspodar	55.7 33.5	53.4 49.6

Samples from four dogs (two in the placebo group and two in the valspodar group) had sufficient material for analysis of ABCB1 and ABCG2 to determine if valspodar specifically reduced the number of ABCB1 ^+^ and ABCG2 ^+^ LPCs in paired pre-and post-treatment samples. There was a quantifiable decrease in the frequency of ABCB1 ^+^ and ABCG2 ^+^ LPCs, but this change was comparable between the two dogs that received valspodar and the two dogs that received placebo ( Table 5 and Supplemental Figures 2A–2D, Dataset 1b).

### Alterations in genome-wide gene expression in lymph nodes from dogs with lymphoma

We examined if the inhibition of ABCB1 activity with valspodar changed genome-wide patterns of gene expression in lymph nodes from dogs in both groups. Paired pre- (Day 0) and post- (Day 4) treatment samples were available from five dogs in the placebo group and from nine dogs in the valspodar group. One additional pre-treatment sample from dogs in each group was available and included in the analysis, making a total of 16 pre-treatment samples and 14 post-treatment samples. Using Cuffdiff analysis, we identified genes with significantly differently expression between treatment groups and between pre- and post-treatment samples ( Supplemental Table 1 and Supplemental Table 2). However, as illustrated by principal component analysis ( Supplemental Figure 3), we were unable to achieve a meaningful separation of groups based on the effects of treatment on genome-wide gene expression.

### Bioavailability of valspodar

The observation that valspodar treatment did not specifically alter the total blood or lymph node LPCs or the frequency of ABCB1 ^+^ and ABCG2 ^+^ LPCs, and that it did not lead to significant changes in genome-wide gene expression of lymph node cells, could be attributed to poor bioavailability. To evaluate this possibility, we examined the purity of the compounded, encapsulated drug and the levels of valspodar in serum samples obtained at Day 4 from seven dogs using LC-MS/MS. Valspodar was undetectable in placebo capsules, and the purity of the compounded capsules was 104% as compared to research grade valspodar.

Valspodar was also undetectable (<5 ng/ml) in dogs that received placebo, but it was present at detectable levels in each of four dogs that received compounded valspodar capsules (34, 63, 375, and 623 ng/ml, respectively). This is equivalent to levels between 0.025 to 0.5 µM on the fourth day of twice-daily administration, which is in the range seen in dogs where valspodar was given at the same dose in an oil-based drinking solution ^14^.

### Clinical responses

Eighteen treated dogs achieved clinical remission, defined as a complete response (disappearance of all evidence of disease with all lymph nodes shrinking to non-pathologic size within the judgment of the evaluator) after the first dose of doxorubicin. One dog in the valspodar group did not achieve clinical remission but survived with stable disease for 428 days. One dog in the placebo group never received doxorubicin and was censored from this analysis. This dog was treated with palliative intent using prednisone only; it failed to achieve remission and died 59 days after diagnosis.

**Figure 6.**
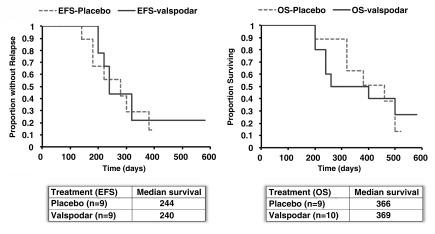
Effect of neoadjuvant valspodar on survival of dogs with large B-cell lymphoma. Kaplan–Meier analysis of event-free survival (top) and overall survival (bottom) in dogs treated with doxorubicin with the addition of neoadjuvant placebo or valspodar. The table below the graphs shows the median event-free and overall survival for each group. Data were analyzed and graphs were assembled using MS Excel.

The time to remission after the initial valspodar treatment ranged from 7 to 106 days (after doxorubicin) in the placebo group, and from 7 to 105 days (after doxorubicin) in the valspodar group (excluding the dog that never achieved remission). There were no differences between groups with reference to the median time to remission, the median (or range) duration of remission, the number of dogs alive at the 180-day milestone, or the number of dogs alive at 500 days ( Table 6). The event-free survival and overall survival times for each group are shown in Figure 6.

**Table 6.** Clinical responses.

	All Dogs	Placebo Group	Valspodar Group
Time to Remission (after Adriamycin) Median (days) Range (days)	21 7-∞	21 7-106	21 7-∞ ^*^
Time to Relapse Median (days) Range (days)	242 136-568	244 136-437	240 137-568
Status at 180 days Alive Dead Lost to follow-up	18 0 1	9 0 0	9 0 1
Survival Median (days) Range (days)	366 185-568	366 191-508	369 185-568
Alive at 500 days	3	1	2

*Excluding dog that did not achieve remission 7-105

### Correlation between ΔLPCs and outcome

**Figure 7.**
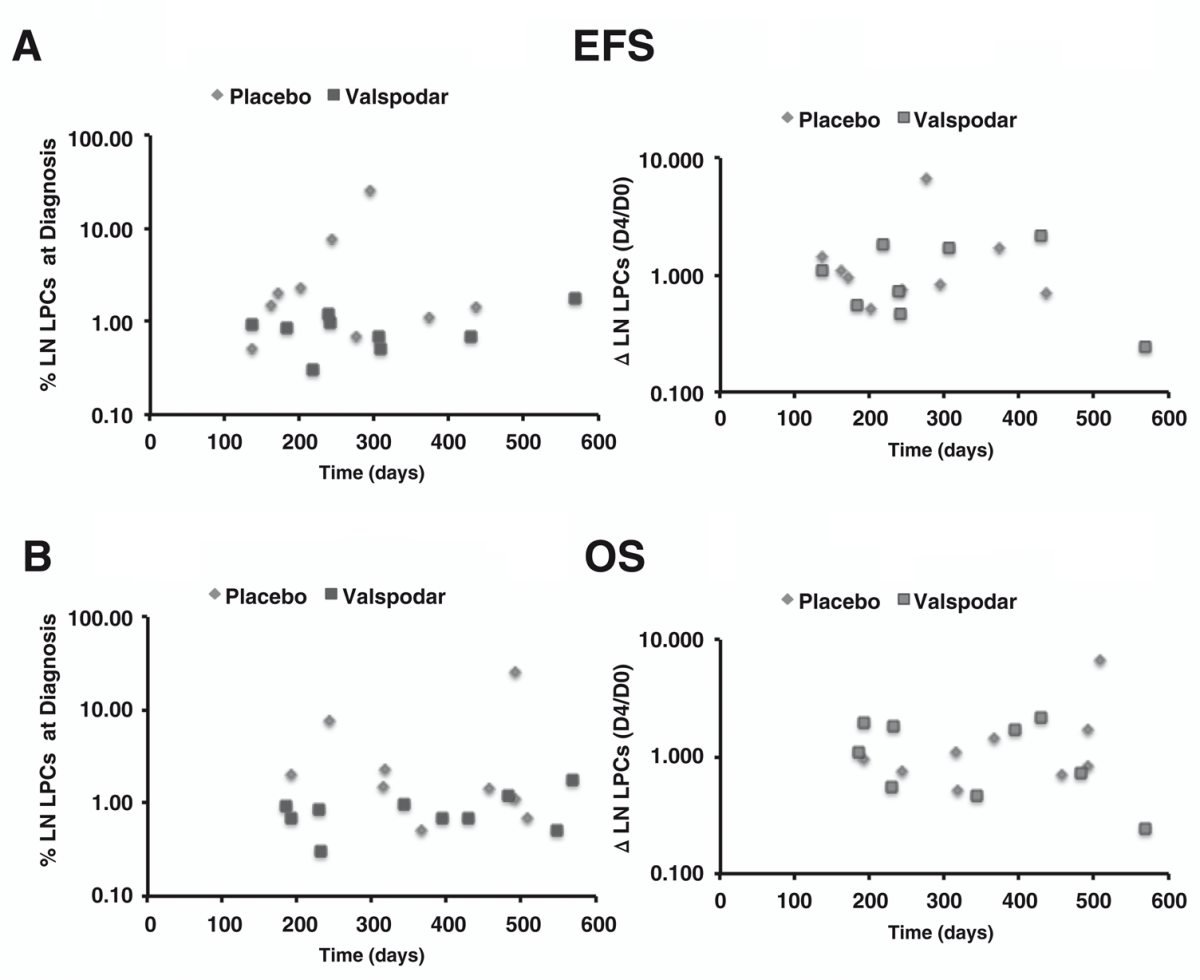
Event-free and overall survival of dogs as a function of lymph node LPCs from dogs with large B-cell lymphoma at diagnosis. ( **A**) Dot plots showing the relationship between the percent of lymph node LPCs at diagnosis and EFS (N=9), and the relative change in LPCs from the time of diagnosis (Day 0) to the fourth day of the neoadjuvant period (Day 4) and EFS (N=8), in days in dogs treated with placebo (N = 9) or with neoadjuvant valspodar. ( **B**) Dot plots showing the same relationships for overall survival (OS, N=9 and N=10 for LPCs at diagnosis and for ΔLPCs, respectively). Data were analyzed and graphs were assembled using MS Excel.

To test the hypothesis that LPCs contribute to disease progression, we examined if there were direct or inverse correlations between the proportion of LPCs at diagnosis and the ΔLPCs with duration of remission as well as with overall survival for dogs in the valspodar and control groups, individually and for all of the dogs in the study. Figure 7A and 7B show scatterplots illustrating no correlations between the proportion of lymph node LPCs at diagnosis and the ΔLPCs (D4/D0), respectively, and event-free survival (duration of remission) and overall survival. The results were similar when we analyzed correlations between the proportion of blood LPCs at diagnosis or ΔLPCs and survival outcomes (data not shown).

Data of pilot study on valspodar in neoadjuvant settings for canine B-cell lymphoma The raw data of this study are grouped in dataset 1a (side population assay) and dataset 1b (expression of ABCB1 and ABCG2 before and after PSC-833 treatment). More details can be found in the text file provided. Click here for additional data file. Copyright: © 2017 Ito D et al. 2017 Data associated with the article are available under the terms of the Creative Commons Zero "No rights reserved" data waiver (CC0 1.0 Public domain dedication).

## Conclusions and discussion

We conducted a double-blinded, placebo controlled study in 20 dogs to determine whether valspodar used in the neoadjuvant setting would sensitize LPCs to doxorubicin and increase the length of remission in dogs with therapy naïve large B-cell lymphoma. Our results confirmed the previous observation from Cagliero *et al.* ^14^ showing that valspodar can be safely administered to dogs twice daily at a dose of 7.5 mg/kg. Furthermore, we verified that CD22 ^+^/CD34 ^+^/CD117 ^+^/CD133 ^+^ LPCs constitute between 0.3 – 2% of lymph node B cells. The narrow conservative gating required due to high inter-sample variability identified the LPC frequencies in peripheral blood at a level of ~1 per 10 ^6^ to 1 per 5 × 10 ^6^ cells. The observation that these cells are virtually undetectable in lymph node samples from healthy dogs, while they exist in a steady state in canine B-cell lymphomas even in the xenotransplantation setting ^6^, suggests that they contribute to the maintenance or propagation of the tumor population. However, we were unable to demonstrate that combined treatment with valspodar and doxorubicin selectively depletes LPCs relative to doxorubicin plus placebo treatment.

Upregulation of ABC transporters is a well-described mechanism of acquired drug resistance in lymphoma and other cancers, making these proteins attractive targets for pharmacologic modulation ^40, 41^. These proteins are transport channels that extrude a variety of compounds, including xenobiotics, from cells. Cells expressing these proteins have been defined functionally as “side populations” based on their ability to exclude fluorescent dyes in flow cytometric assays. The possibility that increased expression of ABCB1 and other transporters was due to selection of cells intrinsically possessing this trait, as opposed to through *de novo* induction of expression, was proposed more than 20 years ago ^42^ and recapitulated most recently in canine lymphomas *in vitro* through drug selection, with expansion of a valspodar-sensitive subclone that had increased expression of ABCB1 and ABCG2 ^43^.

“Side populations” are routinely detectable in canine lymphomas ^44^. In that study, 0.1 to 4% of cells in the canine B-cell lymphoma cell lines GL-1 and 17–71 excluded Hoechst 33342 and expressed detectable levels of ABCB1 and ABCB2. A dye-excluding side population was also variably detectable in five primary lymphomas. GL-1 cells and one of the lymphoma samples expressed a form of ABCB1 with slower electrophoretic mobility, possibly representing the active, phosphorylated form of this transporter ^45^. ABCG2 was expressed ubiquitously in GL-1 cells and in the five primary lymphomas. However, the side population identified by Kim and colleagues was insensitive to verapamil and to fumitremorgin-C ^44^, suggesting that the dye exclusion activity might have been mediated by an ABC transporter distinct from ABCB1 and ABCG2.

The notion that cells expressing ABC transporters can behave like cancer stem cells in lymphomas is not universally accepted. Indeed, the existence of tumor-initiating or tumor-propagating cells (TIC/TPC) or of a hierarchical organization in lymphoid malignancies at all remains a matter of debate ^46^. In acute lymphoblastic leukemias (ALL), models for cells of origin have been proposed, including common hematopoietic progenitors, common lymphoid progenitors, and committed B-lymphoid cells, depending largely upon the molecular subtype of ALL. In preliminary experiments, samples from two human patients with ALL included a subset of CD117 ^+^ cells that were present at a similar frequency to LPCs in canine lymphoma (D. Ito and J. Modiano, unpublished results); however, the functional significance of this finding remains to be determined. The evidence for TIC/TPC in solid lymphomas is even more sparse. Drug-resistant TIC/TPCs were defined in follicular lymphoma using side population assays and increased expression of ABCG2 ^2^. Tumor formation in these cells was limited by an obligate interaction with follicular dendritic cells in the microenvironment niche, which was mediated through the CXCR4 chemokine receptor. TIC/TPC were similarly identified using side population assays in a mouse model of mantle cell lymphoma ^4^, and more recently in human anaplastic lymphoma kinase (ALK)-positive and -negative anaplastic large cell lymphomas ^47^.

Next generation sequencing and genome-wide epigenomic analyses of human DLBCL have revealed a potential mechanism to explain how lymphoid cells might acquire TIC/TPC properties and how this acquisition could be related to the expression of ABC transporters. The gene encoding the enhancer of zeste homolog 2 (EZH2) had gain of function mutations in 7/49 (14%) DLBCL patients sequenced ^48^. EZH2 is a histone methyltransferase that functions as part of the polycomb group complex, which controls the balance between self-renewal and differentiation ^49^. In germinal center (GC) B cells, EZH2 appears to suppress differentiation genes and favor behavior that resembles stem cells ^50^. As in GC DLBCL cells, depletion of EZH2 in Bel/Fu hepatocellular carcinoma cells inhibited proliferation, but in Bel/Fu cells this depletion also increased methylation at the ABCB1 gene, reduced ABCB1 gene and protein expression ^51^, and showed consequent sensitization of these cells to the cytotoxic effects of 5-fluorouracil ^52^. Together, these findings provide a strong rationale for the use of neoadjuvant therapies to sensitize TIC/TPCs in lymphoma using ABC transporter inhibitors, at least in a subset of GC DLBCL.

Nonetheless, clinical results using valspodar in combination with chemotherapy for human patients have been unrewarding. Three large, phase-3 double blinded clinical trials incorporated valspodar into the therapeutic regimen with no improvements in survival outcomes. In the first study ^17^, the experimental group of patients with recurring or refractory multiple myeloma received oral valspodar (4 mg/kg) one day in advance of starting the chemotherapy cycles of vincristine, doxorubicin, and dexamethasone with appropriate dose reductions (50% for vincristine and 22% for doxorubicin). Valspodar was continued daily at the same dose through the sixth day of treatment. More patients had partial responses in the valspodar group, but the difference was not statistically significant and was counteracted by increased toxicity that led to inferior survival, which the investigators attributed to higher bioactive levels of doxorubicinol in the patients receiving valspodar. In the second study ^18^, women with stage IV or suboptimally debulked stage III epithelial ovarian or primary peritoneal cancer were randomly assigned to receive paclitaxel and carboplatin with or without valspodar as first line-chemotherapy. Valspodar was dosed orally at 5 mg/kg every 6 hours for three days with chemotherapy starting one day after valspodar; a reduced dose of paclitaxel was used in the valspodar group to provide equivalent exposure and toxicity between groups. Addition of valspodar to this chemotherapy protocol did not improve time to progression or survival, although it significantly enhanced toxicity. In the third study ^19^ previously untreated patients younger than 60 years old with acute myelogenous leukemia were randomized into treatment groups of cytosine arabinoside, daunorubicin, and etoposide with or without valspodar. Valspodar was administered as a loading dose of 2.8 mg/kg intravenously on the first day followed by a continuous intravenous infusion of 10 mg/kg for 72 hours. The doses of daunorubicin and etoposide were reduced by 2.25-fold and 2.5-fold, respectively to mitigate toxicity. Addition of valspodar did not produce a survival benefit despite increased gastrointestinal toxicity.

It is worth noting, however, that none of these studies evaluated whether tumor cells in general, or cells expressing ABCB1 or other TIC/TPC markers in particular, were sensitized to chemotherapy treatments. Kolitz *et al.* ^19^ indicated that pre-therapy myeloblasts were collected from patients for analysis of ABCB1 expression and modulation of drug efflux *in vitro* by valspodar, but the results of these experiments were not reported. For this study, our intent was to assess the possibility that neoadjuvant treatment with valspodar for 4 days, with maintenance for an additional day after the start of chemotherapy, would sensitize LPCs to the cytotoxic effects of doxorubicin. Our results indicate that LPCs in canine large B-cell lymphoma were heterogeneous regarding the expression of ABCB1 and ABCG2, with slightly fewer positive cells present in the dogs randomized to the placebo group. Such heterogeneity is consistent with previous observations in human lymphoma samples ^3^. The apparent reversal in outcome trends between the placebo and valspodar groups as a function of the percent lymph node B-cell LPCs at diagnosis was intriguing, and while tempered by the small sample size, it suggests this approach merits additional investigation.

Gene expression analysis showed differential changes over time in lymph node samples from dogs treated with placebo and from dogs treated with valspodar. However, the differentially expressed genes were driven by a few samples in each group and there were no global (genome-wide) changes in gene expression that could be attributed to the drug or to time. This is not entirely unexpected, as on average the LPCs comprised less than 3% of the total population of lymph node cells.

The proportion of ABCB1 ^+^ and ABCG2 ^+^ LPCs appeared to decrease in the samples from four dogs during the neoadjuvant period where we could perform the analysis; however, the change was unrelated to valspodar, since a reduction of similar magnitude occurred in the dogs assigned to both the placebo and the valspodar groups. Furthermore, statistically significant differences were not found in either the total number of LPCs or in the duration of remission (or overall survival) between groups of dogs treated with valspodar and placebo.

In fact, the proportion of ABCB1 ^+^ and ABCG2 ^+^ LPCs appeared to decrease in the lymph node samples from four dogs during the neoadjuvant period where we could perform the analysis; however, the change was unrelated to valspodar, since a reduction of similar magnitude occurred in the dogs assigned to both the placebo and the valspodar groups. Furthermore, statistically significant differences were not found in either the total number of LPCs or in the duration of remission (or overall survival) between groups of dogs treated with valspodar and placebo. Similarly, our data suggest that neoadjuvant valspodar had little effect to sensitize peripheral blood LPCs to doxorubicin. Yet, the morphologic variability in peripheral blood leukocytes and noise in the system, both among dogs as well as within dogs sampled at different time points, confounded our attempts to create a robust strategy for identifying blood LPCs (rare events) with a high degree of certainty. The preponderance of evidence does not support an effect of valspodar to sensitize LPCs to chemotherapy. However, we must consider that the sample size ( *i.e.*, the number of dogs) could be insufficient to demonstrate the effect given the extremely low frequency of cells with LPC phenotype and the pronounced inter-sample variability, so we must consider the data from peripheral blood LPCs inconclusive. Nonetheless, our results suggest that LPC cell enumeration is technically possible, and future attempts to document changes in proportions of LPCs will need to be paired with improved and rigorous quality control measures and a sufficiently large sample size to minimize or manage this variability.

It is worth noting that the duration of remission and the overall survival of dogs in this study slightly exceeded the expectations based on previously published results using single agent doxorubicin ^36^. This could be attributed to improved management of cancer patients over time, but it also could be due to recruitment of a relatively uniform population of dogs based on clinical and pathologic criteria ^23^. The latter possibility highlights the benefits of study designs that narrow disease heterogeneity, particularly for canine lymphoma where each disease entity in this complex should be considered as an individual condition.

There are several possible explanations for the absence of clinical improvement in dogs receiving valspodar vs. placebo. First, it is possible that this treatment would be most effective against a specific subset of DLBCL, such as EZH2-mutated GC DLBCL. It has been challenging to separate canine DLBCLs into activated B-cell (ABC) type and GC-type DLBCL ^11, 53^, although one study suggested canine DLBCL might be more similar to human ABC type DLBCL ^54^. Second, the study was designed to address chemosensitization of LPCs by valspodar, and the sample size was not powered to reveal if this protocol would significantly improve survival outcomes. Based on our results, we estimate that a clinical trial where we could detect a doubling of the median overall survival (from 12 months to 24 months) in dogs receiving neoadjuvant valspodar would require 35 dogs each in the treatment and in the placebo arms.

We confirmed absorption and bioavailability of the drug on the fourth day of administration, and we showed that the drug was able to fully inhibit ABC transporter activity in a side population assay even at the lowest dose detected. However, the levels of valspodar required for sustained, active inhibition of ABC transporter activity *in vivo* have not been conclusively established. For example, when valspodar (50 mg/kg) and paclitaxel (10 mg/kg) were administered concurrently to mice through the oral route, they passed rapidly through the stomach and reached the intestine together, but showed enhanced uptake and plasma levels for paclitaxel ^55^. In rats, oral valspodar was absorbed rapidly and had excellent bioavailability with low hepatic extraction ^56^. In human patients with chemotherapy-resistant multiple myeloma, a dose escalation study showed similar pharmacokinetic properties. Orally administered valspodar combined with doxorubicin, vincristine and dexamethasone led to a doubling of the area under the curve for doxorubicin levels in the plasma and reduced its clearance by half ^16^. The concentration of valspodar in serum increased proportionately with a dose of up to 15 mg/kg/day, although it reached a maximum effectiveness level vis-à-vis increasing plasma doxorubicin at 5 mg/kg/day where the median trough and peak levels (of valspodar) were 461 ng/ml and 1134 ng/ml, respectively. The treatment regimen was associated with increased toxicity and required dose reduction in more than 50% of the patients (13/22). Yet, 14 of the patients treated had either a partial response or stable disease, and ABCB1 expression in bone marrow plasma cells was reduced in four of the five responding patients examined.

In another study, valspodar was administered concurrently with doxorubicin to 31 cancer patients using an intravenous loading dose of 1–2 mg/kg and a continuous dose of 1–10 mg/kg over 24 hours. Doxorubicin was given immediately at the end of the loading dose, and the treatment was repeated every 21 days until there was disease progression or unacceptable toxicity ^15^. As noted in the Sonneveld study ^16^, patients receiving valspodar showed a significantly increased area under the curve for doxorubicin, with a 50% shortening of doxorubicin clearance as compared to controls. The steady-state concentrations of valspodar over the time of continuous administration ranged from 190 ng/ml to 1383 ng/ml with unchanged rates of clearance, and serum from treated patients contained sufficiently high levels of valspodar to inhibit ABCB1 activity in an *in vitro* bioassay. Dose-limiting toxicities were observed only in patients treated with the highest dose of valspodar (2 mg/kg loading dose and 10 mg/kg continuous dose) and 50 mg/kg doxorubicin. One patient (ovarian cancer) had a partial response, but none of the patients in this trial had non-Hodgkin lymphoma ^15^.

The effective serum concentrations and positive bioassay results in these studies are in contrast to those in another series of experiments showing that the concentration required to inhibit ABC transporter activity *in vitro* under complete serum conditions (cells cultured in 100% fetal bovine serum) is almost a full order of magnitude (8–9 times) higher than the plasma concentrations achieved in clinical trials, probably due to binding of valspodar by serum lipoproteins ^57^. Among the compounds examined, daunorubicin was the most relevant. In 100% serum, the half maximal concentration of valspodar required to inhibit ABCB1-mediated daunorubicin transport was approximately 1.5 µM (or approximately 1800 ng/ml), which is close to the peak levels achievable using continuous infusions ^15^ and almost 3-fold higher than the levels we measured in our study.

It also is possible that inhibiting ABCB1 and ABCG2 in LPCs is insufficient to ablate the population. In our study, 30% to 90% of lymph node LPCs did not express ABCB1 or ABCG2. In addition, the variable sensitivity to verapamil and other ABC transporter inhibitors by LPCs and side population cells in leukemia and lymphoma suggests that these cells might rely on alternative mechanisms of drug export and/or drug resistance. Still, it has been shown that clinically relevant anti-lymphoma immunotherapies including rituximab ^58^ and anti-CD19 antibodies ^59^ induce ABCB1 to translocate out of lipid rafts, reducing its ability to extrude chemotherapy agents such as vincristine and doxorubicin and increasing the chemosensitivity of drug-resistant lymphoma cell lines. We propose that the totality of data continues to support the rationale for implementing treatment approaches for non-Hodgkin lymphoma that target ABCB1 and ABCG2 in the neoadjuvant or the adjuvant settings. These treatments might be most effective for patients with tumors that do not respond to other targeted agents, such as those diagnosed with EZH2-mutant GC DLBCL. Thus, additional work and diligently crafted clinical trials, as well as creative animal models of induced and spontaneous disease, will be needed to establish the significance of LPCs in the pathogenesis of lymphoid malignancies and the potential to improve patient outcomes by targeting the ABC transporter-enriched and the ABC transporter-deficient subsets of these cell populations.

## Data availability

The data referenced by this article are under copyright with the following copyright statement: Copyright: © 2017 Ito D et al.

Data associated with the article are available under the terms of the Creative Commons Zero "No rights reserved" data waiver (CC0 1.0 Public domain dedication). 

*F1000Research*: Dataset 1. Data of pilot study on valspodar in neoadjuvant settings for canine B-cell lymphoma, 10.5256/f1000research.6055.d42897 ^60^

RNA sequencing FASTQ files and processed data files are available through the National Center for Bioinformatics (GSE93516).

## Supplementary materials

**Supplemental Figure 1. Identification of putative LPCs in the peripheral blood of dogs with diffuse large B-cell lymphoma.** Two-dimensional scatter plots depicting gating strategies used to identify putative LPCs in peripheral blood. Panels A and B depict data from two separate dogs on Day 1. Panels **B** through **D** depict data from a single dog on Days 1 ( **B**), 4 ( **C**), and 11 ( **D**). Cells co-expressing CD22 (520 BP Area (FITC)) and the “Progenitor mix” of CD133, CD117, and CD34 (575 BP Area (PE)) were identified in the peripheral blood of all dogs. However, the significant variability in light scatter parameters noted between dogs (Panels **A** and **B**), as well as within individual dogs at the three sampling time points (Panels **B** through **D**), limits the ability to interpret these data with a high degree of confidence.

**Supplemental Figure 2. Expression of ABCB1 and ABCG2 in lymph node LPCs from dogs with large B-cell lymphoma at diagnosis and on the fourth day of the neoadjuvant period.** Samples from two dogs in each group were available for analysis of ABCB1 and ABCG2 expression in LPCs prior to (Day 0) and on the fourth day (Day 4) of neoadjuvant treatment. Live lymphocytes were gated based on light scatter properties and exclusion of 7-AAD. T cells were excluded based on CD5 staining; progenitor cells were gated based on expression of CD34, CD117, and CD133 as described in Materials and methods. Dye exclusion was measured as described in Figure 2. ( **A**) ABCB1 and ABCG2 expression at the time of diagnosis (Day 0) in two dogs treated with valspodar (MN02 and MN10). ( **B**) ABCB1 and ABCG2 expression on the fourth day of neoadjuvant treatment (Day 4) in two dogs treated with valspodar (MN02 and MN10). ( **C**) ABCB1 and ABCG2 expression at the time of diagnosis (Day 0) in two dogs treated with placebo (MN05 and MN09). ( **D**) ABCB1 and ABCG2 expression on the fourth day of neoadjuvant treatment (Day 4) in two dogs treated with placebo (MN05 and MN09). Data were analyzed and dot plots were created in FlowJo.

**Supplemental Figure 3. Principal Components Analysis (PCA).** The FPKM values for the genes were log-transformed using log2 (FPKM+offset) with an offset of 0.1. A filtering criterion of a standard deviation estimate >0.1 for each gene (across samples) was used to obtain 11, 120 expressed genes. The built-in R function *prcomp* was used to perform PCA with the filtered log2(FPKM + 0.1) values in each sample. The first component was plotted against the second component with the ‘ggplot2’ and ‘ggfortify’ packages in Rstudio.

.

**Supplemental Table 1. Differentially expressed genes (DGE) from Cuffdiff analysis between pre and post placebo treatment groups.** Cuffdiff analysis was based on FPKM (fragments per kilobase of transcript per million reads and false discovery rate, FDR q < 0.05). Pre-treatment samples were given the “sample_1” label and post-treatment samples were given the “sample_2” label for testing for group differences. An “OK” test status means that the test was successful. The “value_1” is the Cuffdiff computed FPKM of the sample_1 group whereas the “value_2” is the Cuffdiff computed FPKM of the sample_2 group. The log2 (fold_change) is the (base 2) log fold change of FPKM _value_1_/FPKM _value_1_. The test statistic is the value of the test statistic used to compute significance of the observed change in FPKM. The q value is the FDR-adjusted p-value and “yes” in the significance column means that the p value was less than the q value after Benjamini-Hochberg correction for multiple testing.

**Supplemental Table 2. Differentially expressed genes (DGE) from Cuffdiff analysis between pre and post valspodar treatment groups.** Cuffdiff analysis was based on FPKM (fragments per kilobase of transcript per million reads and false discovery rate, FDR q < 0.05). The locus is the genomic coordinates for the gene that was tested. Pre-treatment samples were given the “sample_1” label and post-treatment samples were given the “sample_2” label for testing for group differences. An “OK” test status means that the test was successful. The “value_1” is the Cuffdiff computed FPKM of the sample_1 group whereas the “value_2” is the Cuffdiff computed FPKM of the sample_2 group. The log2 (fold_change) is the (base 2) log fold change of FPKM _value_1_/FPKM _value_1_. The test statistic is the value of the test statistic used to compute significance of the observed change in FPKM. The q value is the FDR-adjusted p-value and “yes” in the significance column means that the p value was less than the q value after Benjamini-Hochberg correction for multiple testing.

## References

[1] Nguyen LV Vanner R Dirks P: Cancer stem cells: an evolving concept. *Nat Rev Cancer.* 2012;12(2):133–43. 10.1038/nrc3184 22237392

[2] Lee CG Das B Lin TL: A rare fraction of drug-resistant follicular lymphoma cancer stem cells interacts with follicular dendritic cells to maintain tumourigenic potential. *Br J Haematol.* 2012;158(1):79–90. 10.1111/j.1365-2141.2012.09123.x 22509798 PMC3374069

[3] Lee MR Ju HJ Kim BS: Isolation of side population cells in B-cell non-Hodgkin's lymphomas. *Acta Haematol.* 2013;129(1):10–7. 10.1159/000341284 22964907

[4] Vega F Davuluri Y Cho-Vega JH: Side population of a murine mantle cell lymphoma model contains tumour-initiating cells responsible for lymphoma maintenance and dissemination. *J Cell Mol Med.* 2010;14(6B):1532–45. 10.1111/j.1582-4934.2009.00865.x 19656242 PMC3829019

[5] Wang Y Liu Y Malek SN: Targeting HIF1α eliminates cancer stem cells in hematological malignancies. *Cell Stem Cell.* 2011;8(4):399–411. 10.1016/j.stem.2011.02.006 21474104 PMC3084595

[6] Ito D Endicott MM Jubala CM: A tumor-related lymphoid progenitor population supports hierarchical tumor organization in canine B-cell lymphoma. *J Vet Intern Med.* 2011;25(4):890–6. 10.1111/j.1939-1676.2011.0756.x 21777289 PMC4993164

[7] Ito D Frantz AM Modiano JF: Canine lymphoma as a comparative model for human non-Hodgkin lymphoma: recent progress and applications. *Vet Immunol Immunopathol.* 2014;159(3–4):192–201. 10.1016/j.vetimm.2014.02.016 24642290 PMC4994713

[8] Donnenberg VS Donnenberg AD: Multiple drug resistance in cancer revisited: the cancer stem cell hypothesis. *J Clin Pharmacol.* 2005;45(8):872–7. 10.1177/0091270005276905 16027397

[9] Gottesman MM Fojo T Bates SE: Multidrug resistance in cancer: role of ATP-dependent transporters. *Nat Rev Cancer.* 2002;2(1):48–58. 10.1038/nrc706 11902585

[10] Ueda K Cardarelli C Gottesman MM: Expression of a full-length cDNA for the human "MDR1" gene confers resistance to colchicine, doxorubicin, and vinblastine. *Proc Natl Acad Sci U S A.* 1987;84(9):3004–8. 10.1073/pnas.84.9.3004 3472246 PMC304789

[11] Frantz AM Sarver AL Ito D: Molecular profiling reveals prognostically significant subtypes of canine lymphoma. *Vet Pathol.* 2013;50(4):693–703. 10.1177/0300985812465325 23125145 PMC4683027

[12] Nobili S Landini I Giglioni B: Pharmacological strategies for overcoming multidrug resistance. *Curr Drug Targets.* 2006;7(7):861–79. 10.2174/138945006777709593 16842217

[13] Tai HL: Technology evaluation: Valspodar, Novartis AG. *Curr Opin Mol Ther.* 2000;2(4):459–67. 11249778

[14] Cagliero E Ferracini R Morello E: Reversal of multidrug-resistance using Valspodar (PSC 833) and doxorubicin in osteosarcoma. *Oncol Rep.* 2004;12(5):1023–31. 10.3892/or.12.5.1023 15492788

[15] Minami H Ohtsu T Fujii H: Phase I study of intravenous PSC-833 and doxorubicin: reversal of multidrug resistance. *Jpn J Cancer Res.* 2001;92(2):220–30. 10.1111/j.1349-7006.2001.tb01085.x 11223552 PMC5926698

[16] Sonneveld P Marie JP Huisman C: Reversal of multidrug resistance by SDZ PSC 833, combined with VAD (vincristine, doxorubicin, dexamethasone) in refractory multiple myeloma. A phase I study. *Leukemia.* 1996;10(11):1741–50. 8892677

[17] Friedenberg WR Rue M Blood EA: Phase III study of PSC-833 (valspodar) in combination with vincristine, doxorubicin, and dexamethasone (valspodar/VAD) versus VAD alone in patients with recurring or refractory multiple myeloma (E1A95): a trial of the Eastern Cooperative Oncology Group. *Cancer.* 2006;106(4):830–8. 10.1002/cncr.21666 16419071

[18] Lhommé C Joly F Walker JL: Phase III study of valspodar (PSC 833) combined with paclitaxel and carboplatin compared with paclitaxel and carboplatin alone in patients with stage IV or suboptimally debulked stage III epithelial ovarian cancer or primary peritoneal cancer. *J Clin Oncol.* 2008;26(16):2674–82. 10.1200/JCO.2007.14.9807 18509179

[19] Kolitz JE George SL Marcucci G: P-glycoprotein inhibition using valspodar (PSC-833) does not improve outcomes for patients younger than age 60 years with newly diagnosed acute myeloid leukemia: Cancer and Leukemia Group B study 19808. *Blood.* 2010;116(9):1413–21. 10.1182/blood-2009-07-229492 20522709 PMC2938834

[20] Ito D Brewer S Modiano JF: Development of a novel anti-canine CD20 monoclonal antibody with diagnostic and therapeutic potential. *Leuk Lymphoma.* 2015;56(1):219–225. 10.3109/10428194.2014.914193 24724777 PMC5002357

[21] Ito D Frantz AM Williams C: CD40 ligand is necessary and sufficient to support primary diffuse large B-cell lymphoma cells in culture: a tool for *in vitro* preclinical studies with primary B-cell malignancies. *Leuk Lymphoma.* 2012;53(7):1390–8. 10.3109/10428194.2011.654337 22229753 PMC3727651

[22] Gorden BH Kim JH Sarver AL: Identification of three molecular and functional subtypes in canine hemangiosarcoma through gene expression profiling and progenitor cell characterization. *Am J Pathol.* 2014;184(4):985–95. 10.1016/j.ajpath.2013.12.025 24525151 PMC3969990

[23] Valli VE San Myint M Barthel A: Classification of canine malignant lymphomas according to the World Health Organization criteria. *Vet Pathol.* 2011;48(1):198–211. 10.1177/0300985810379428 20861499

[24] Mealey KL: Therapeutic implications of the MDR-1 gene. *J Vet Pharmacol Ther.* 2004;27(5):257–64. 10.1111/j.1365-2885.2004.00607.x 15500562

[25] Mealey KL Bentjen SA Gay JM: Ivermectin sensitivity in collies is associated with a deletion mutation of the mdr1 gene. *Pharmacogenetics.* 2001;11(8):727–33. 10.1097/00008571-200111000-00012 11692082

[26] Chan AW Tetzlaff JM Altman DG: SPIRIT 2013 statement: defining standard protocol items for clinical trials. *Ann Intern Med.* 2013;158(3):200–7. 10.7326/0003-4819-158-3-201302050-00583 23295957 PMC5114123

[27] Jubala CM Wojcieszyn JW Valli VE: CD20 expression in normal canine B cells and in canine non-Hodgkin lymphoma. *Vet Pathol.* 2005;42(4):468–76. 10.1354/vp.42-4-468 16006606

[28] Vail DM: Veterinary Co-operative Oncology Group - Common Terminology Criteria for Adverse Events (VCOG-CTCAE) following chemotherapy or biological antineoplastic therapy in dogs and cats v1.0. *Vet Comp Oncol.* 2004;2(4):195–213. 10.1111/j.1476-5810.2004.0053b.x 19379294

[29] Khammanivong A Gorden BH Frantz AM: Identification of drug-resistant subpopulations in canine hemangiosarcoma. *Vet Comp Oncol.* 2014. 10.1111/vco.12114 25112808 PMC5712213

[30] Gorden BH Saha J Khammanivong A: Lysosomal drug sequestration as a mechanism of drug resistance in vascular sarcoma cells marked by high CSF-1R expression. *Vasc Cell.* 2014;6:20. 10.1186/2045-824X-6-20 25295160 PMC4188569

[31] Bolger AM Lohse M Usadel B: Trimmomatic: a flexible trimmer for Illumina sequence data. *Bioinformatics.* 2014;30(15):2114–20. 10.1093/bioinformatics/btu170 24695404 PMC4103590

[32] Kim D Langmead B Salzberg SL: HISAT: a fast spliced aligner with low memory requirements. *Nat Methods.* 2015;12(4):357–60. 10.1038/nmeth.3317 25751142 PMC4655817

[33] Li H Handsaker B Wysoker A: The Sequence Alignment/Map format and SAMtools. *Bioinformatics.* 2009;25(16):2078–19. 10.1093/bioinformatics/btp352 19505943 PMC2723002

[34] Trapnell C Roberts A Goff L: Differential gene and transcript expression analysis of RNA-seq experiments with TopHat and Cufflinks. *Nat Protoc.* 2012;7(3):562–78. 10.1038/nprot.2012.016 22383036 PMC3334321

[35] Vail DM Michels GM Khanna C: Response evaluation criteria for peripheral nodal lymphoma in dogs (v1.0)--a Veterinary Cooperative Oncology Group (VCOG) consensus document. *Vet Comp Oncol.* 2010;8(1):28–37. 10.1111/j.1476-5829.2009.00200.x 20230579

[36] Chun R: Lymphoma: which chemotherapy protocol and why? *Top Companion Anim Med.* 2009;24(3):157–62. 10.1053/j.tcam.2009.03.003 19732735

[37] Binkhathlan Z Somayaji V Brocks DR: Development of a liquid chromatography-mass spectrometry (LC/MS) assay method for the quantification of PSC 833 (Valspodar) in rat plasma. *J Chromatogr B Analyt Technol Biomed Life Sci.* 2008;869(1–2):31–7. 10.1016/j.jchromb.2008.05.003 18514043

[38] Modiano JF Breen M Avery AC: Breed Specific Canine Lymphoproliferative Diseases. In: Ostrander EA, Giger U, Lindblad-Toh K editors. *The Dog and its Genome.*Cold Spring Harbor: CSH Press;2005 Reference Source

[39] Modiano JF Breen M Burnett RC: Distinct B-cell and T-cell lymphoproliferative disease prevalence among dog breeds indicates heritable risk. *Cancer Res.* 2005;65(13):5654–61. 10.1158/0008-5472.CAN-04-4613 15994938

[40] Tan B Piwnica-Worms D Ratner L: Multidrug resistance transporters and modulation. *Curr Opin Oncol.* 2000;12(5):450–8. 10.1097/00001622-200009000-00011 10975553

[41] Modok S Mellor HR Callaghan R: Modulation of multidrug resistance efflux pump activity to overcome chemoresistance in cancer. *Curr Opin Pharmacol.* 2006;6(4):350–4. 10.1016/j.coph.2006.01.009 16690355

[42] Rodriguez C Commes T Robert J: Expression of P-glycoprotein and anionic glutathione S-transferase genes in non-Hodgkin's lymphoma. *Leuk Res.* 1993;17(2):149–56. 10.1016/0145-2126(93)90060-X 8094105

[43] Zandvliet M Teske E Schrickx JA: Multi-drug resistance in a canine lymphoid cell line due to increased P-glycoprotein expression, a potential model for drug-resistant canine lymphoma. *Toxicol In Vitro.* 2014;28(8):1498–506. 10.1016/j.tiv.2014.06.004 24975508

[44] Kim MC D'Costa S Suter S: Evaluation of a side population of canine lymphoma cells using Hoechst 33342 dye. *J Vet Sci.* 2013;14(4):481–6. 10.4142/jvs.2013.14.4.481 23820219 PMC3885743

[45] Idriss HT Hannun YA Boulpaep E: Regulation of volume-activated chloride channels by P-glycoprotein: phosphorylation has the final say! *J Physiol.* 2000;524(Pt 3):629–36. 10.1111/j.1469-7793.2000.00629.x 10790147 PMC2269906

[46] Bernt KM Armstrong SA: Leukemia stem cells and human acute lymphoblastic leukemia. *Semin Hematol.* 2009;46(1):33–8. 10.1053/j.seminhematol.2008.09.010 19100366 PMC4031465

[47] Moti N Malcolm T Hamoudi R: Anaplastic large cell lymphoma-propagating cells are detectable by side population analysis and possess an expression profile reflective of a primitive origin. *Oncogene.* 2015;34(14):1843–52. 10.1038/onc.2014.112 24814516

[48] Lohr JG Stojanov P Lawrence MS: Discovery and prioritization of somatic mutations in diffuse large B-cell lymphoma (DLBCL) by whole-exome sequencing. *Proc Natl Acad Sci U S A.* 2012;109(10):3879–84. 10.1073/pnas.1121343109 22343534 PMC3309757

[49] Lund K Adams PD Copland M: EZH2 in normal and malignant hematopoiesis. *Leukemia.* 2014;28(1):44–9. 10.1038/leu.2013.288 24097338

[50] Velichutina I Shaknovich R Geng H: EZH2-mediated epigenetic silencing in germinal center B cells contributes to proliferation and lymphomagenesis. *Blood.* 2010;116(24):5247–55. 10.1182/blood-2010-04-280149 20736451 PMC3012542

[51] Zhang Y Liu G Lin C: Silencing the EZH2 gene by RNA interference reverses the drug resistance of human hepatic multidrug-resistant cancer cells to 5–Fu. *Life Sci.* 2013;92(17–19):896–902. 10.1016/j.lfs.2013.03.010 23562851

[52] Tang B Zhang Y Liang R: RNAi-mediated EZH2 depletion decreases MDR1 expression and sensitizes multidrug-resistant hepatocellular carcinoma cells to chemotherapy. *Oncol Rep.* 2013;29(3):1037–42. 10.3892/or.2013.2222 23291714

[53] Mudaliar MA Haggart RD Miele G: Comparative gene expression profiling identifies common molecular signatures of NF-κB activation in canine and human diffuse large B cell lymphoma (DLBCL). *PLoS One.* 2013;8(9):e72591. 10.1371/journal.pone.0072591 24023754 PMC3762807

[54] Richards KL Motsinger-Reif AA Chen HW: Gene profiling of canine B-cell lymphoma reveals germinal center and postgerminal center subtypes with different survival times, modeling human DLBCL. *Cancer Res.* 2013;73(16):5029–39. 10.1158/0008-5472.CAN-12-3546 23783577 PMC3755352

[55] Bardelmeijer HA Ouwehand M Beijnen JH: Efficacy of novel P-glycoprotein inhibitors to increase the oral uptake of paclitaxel in mice. *Invest New Drugs.* 2004;22(3):219–29. 10.1023/B:DRUG.0000026248.45084.21 15122069

[56] Binkhathlan Z Hamdy DA Brocks DR: Pharmacokinetics of PSC 833 (valspodar) in its Cremophor EL formulation in rat. *Xenobiotica.* 2010;40(1):55–61. 10.3109/00498250903331056 19903013

[57] Smith AJ Mayer U Schinkel AH: Availability of PSC833, a substrate and inhibitor of P-glycoproteins, in various concentrations of serum. *J Natl Cancer Inst.* 1998;90(15):1161–6. 10.1093/jnci/90.15.1161 9701366

[58] Ghetie MA Crank M Kufert S: Rituximab but not other anti-CD20 antibodies reverses multidrug resistance in 2 B lymphoma cell lines, blocks the activity of P-glycoprotein (P-gp), and induces P-gp to translocate out of lipid rafts. *J Immunother.* 2006;29(5):536–44. 10.1097/01.cji.0000211307.05869.6c 16971809

[59] Ghetie MA Marches R Kufert S: An anti-CD19 antibody inhibits the interaction between P-glycoprotein (P-gp) and CD19, causes P-gp to translocate out of lipid rafts, and chemosensitizes a multidrug-resistant (MDR) lymphoma cell line. *Blood.* 2004;104(1):178–83. 10.1182/blood-2003-12-4255 15001473

[60] Ito D Childress MO Mason NJ: Data of pilot study on valspodar in neoadjuvant settings for canine B-cell lymphoma. *F1000Research.* 2015 Data Source

