# Supplementary material for: A double blinded, placebo-controlled pilot study to examine reduction of CD34 +/CD117 +/CD133 + lymphoma progenitor cells and duration of remission induced by neoadjuvant valspodar in dogs with large B-cell lymphoma
Source: F1000Res. 2017 Apr 18;4:42. Originally published 2015 Feb 11. [Version 3] doi: 10.12688/f1000research.6055.3 (PMC5357040; doi:10.12688/f1000research.6055.3)
Supplement: Supplementary file 2 [file f1000research-4-12329-s0001.tgz › 30aef66b-8d2d-448f-a25c-b07751e666db.pptx]

## Slide 1
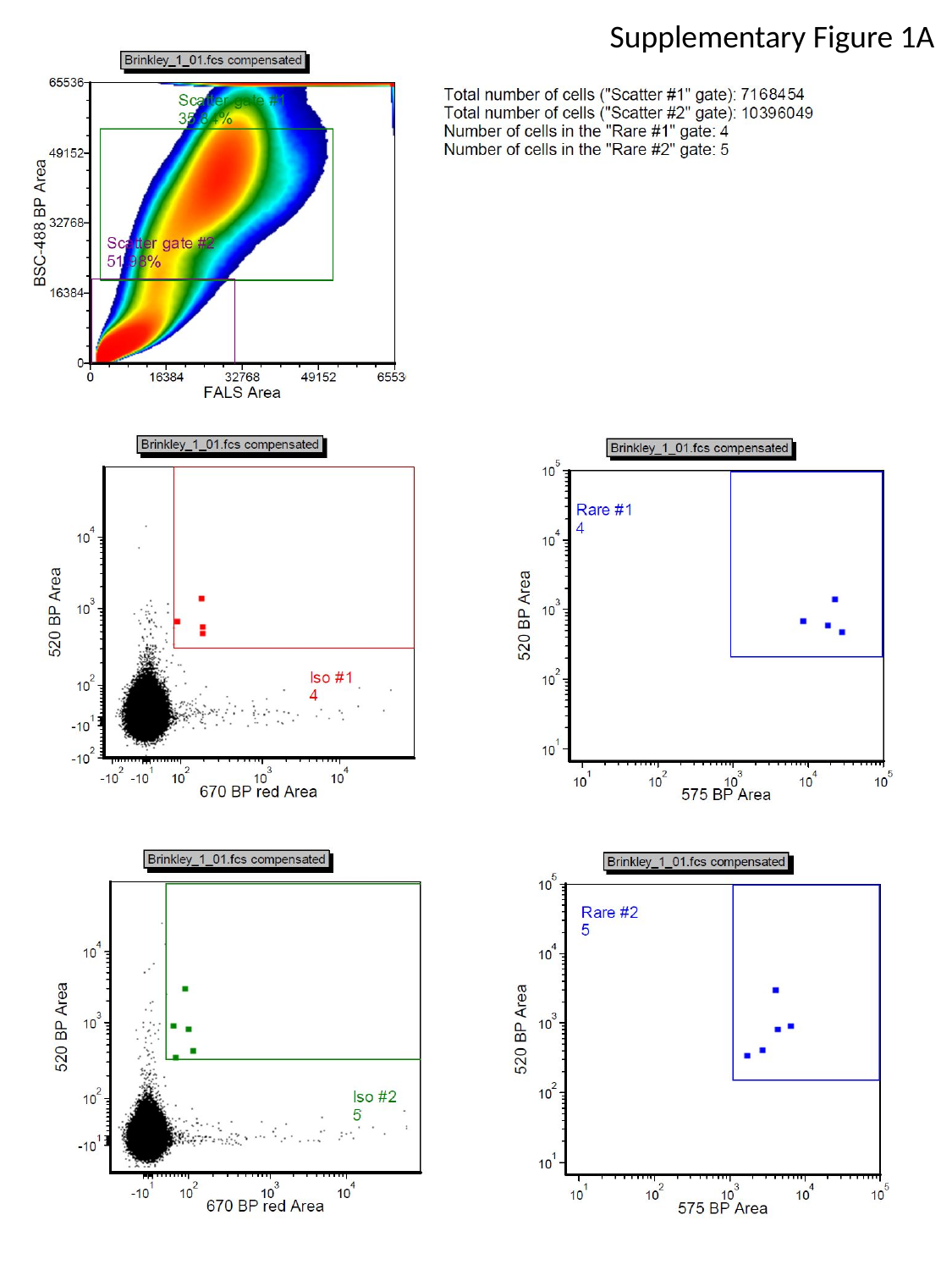

Supplementary Figure 1A

## Slide 2
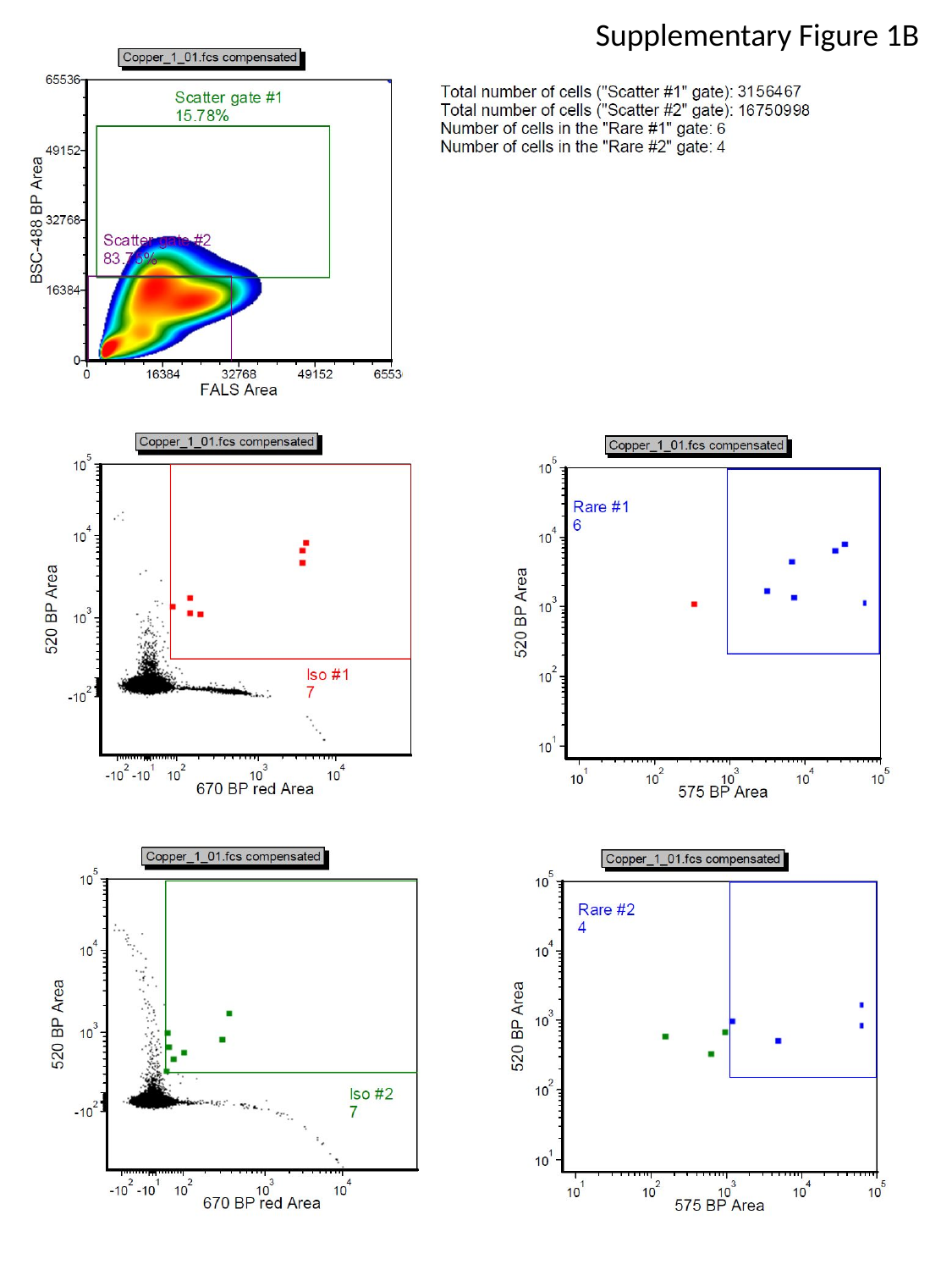

Supplementary Figure 1B

## Slide 3
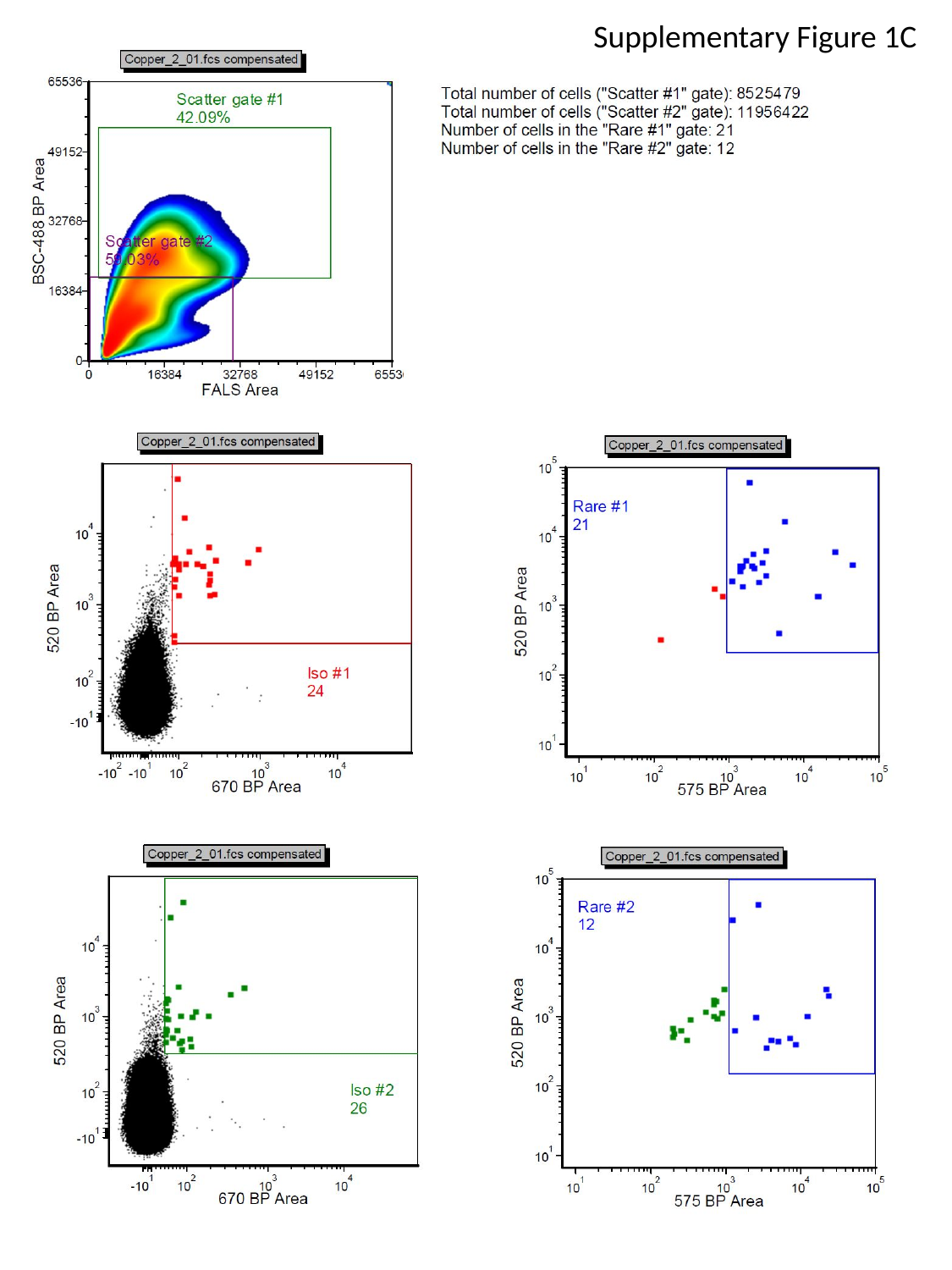

Supplementary Figure 1C

## Slide 4
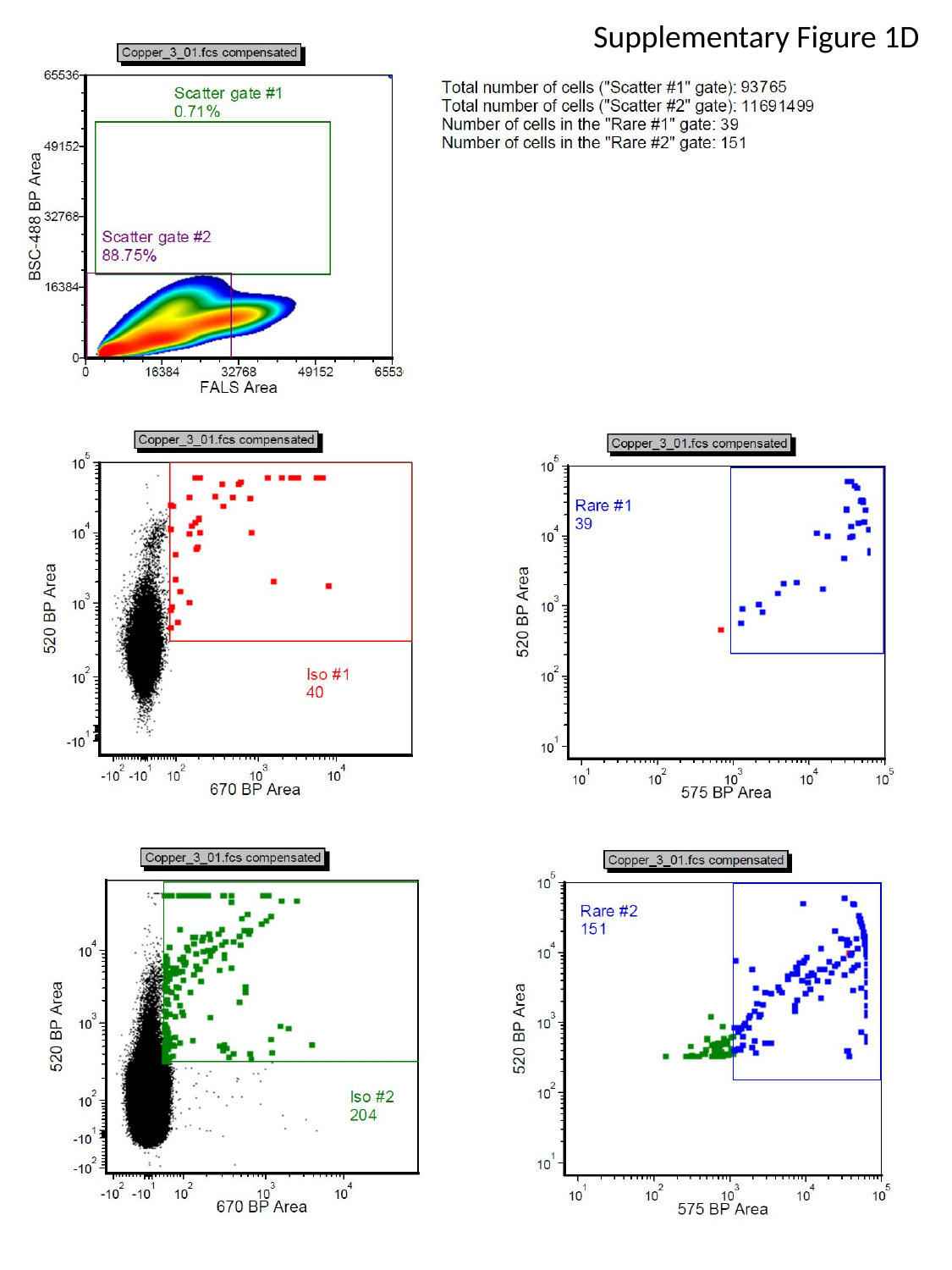

Supplementary Figure 1D

## Slide 5
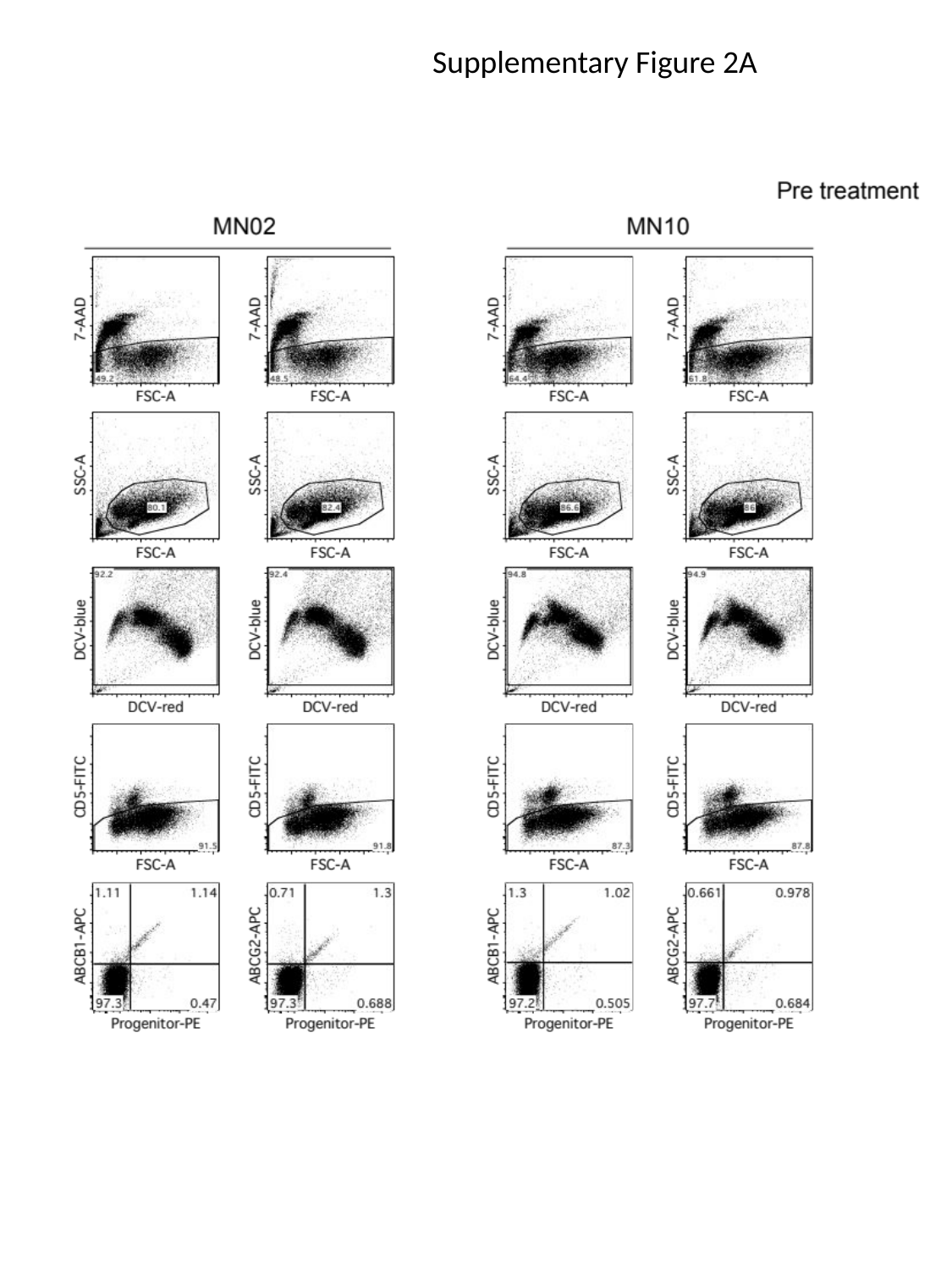

Supplementary Figure 2A

## Slide 6
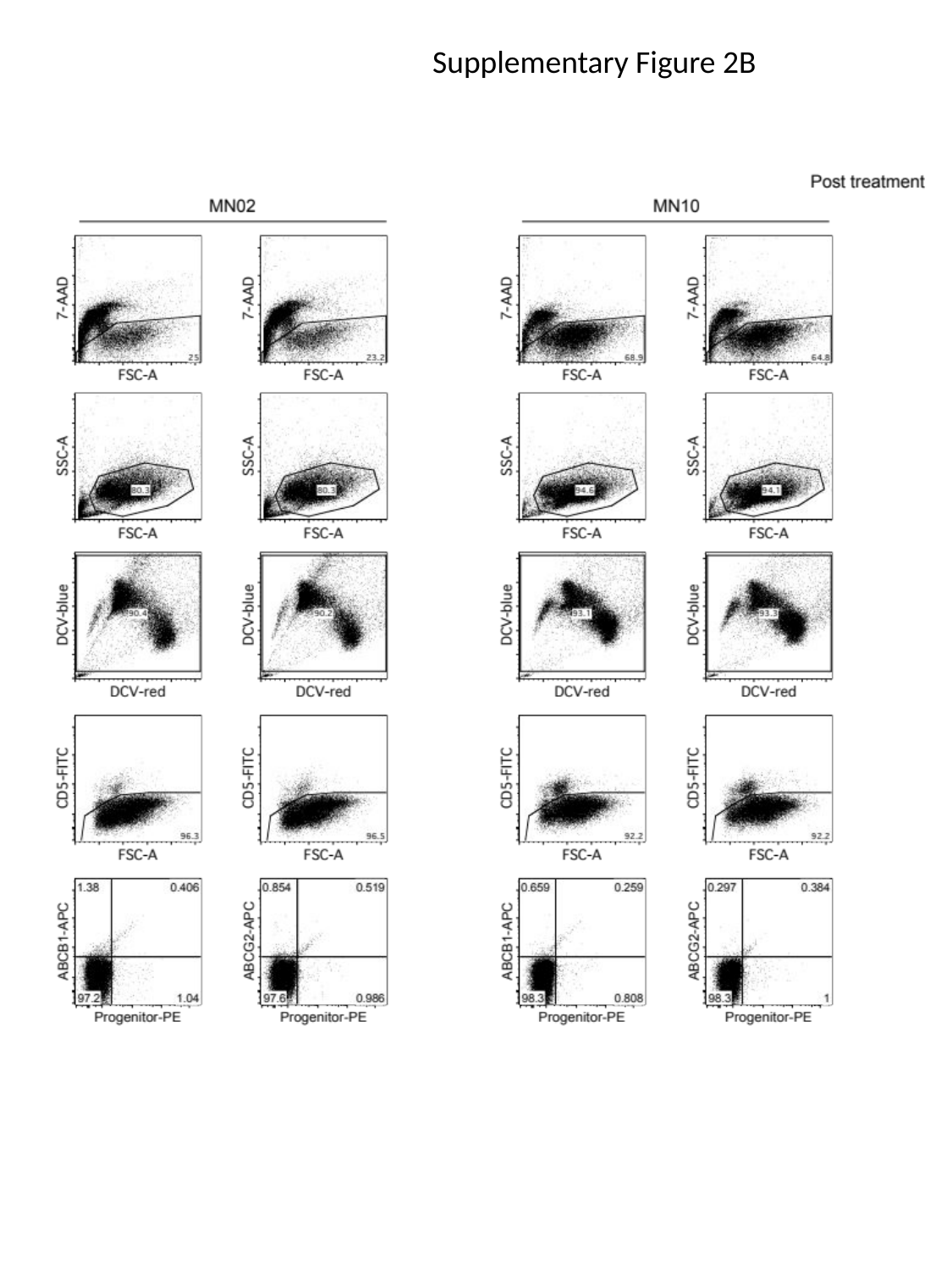

Supplementary Figure 2B

## Slide 7
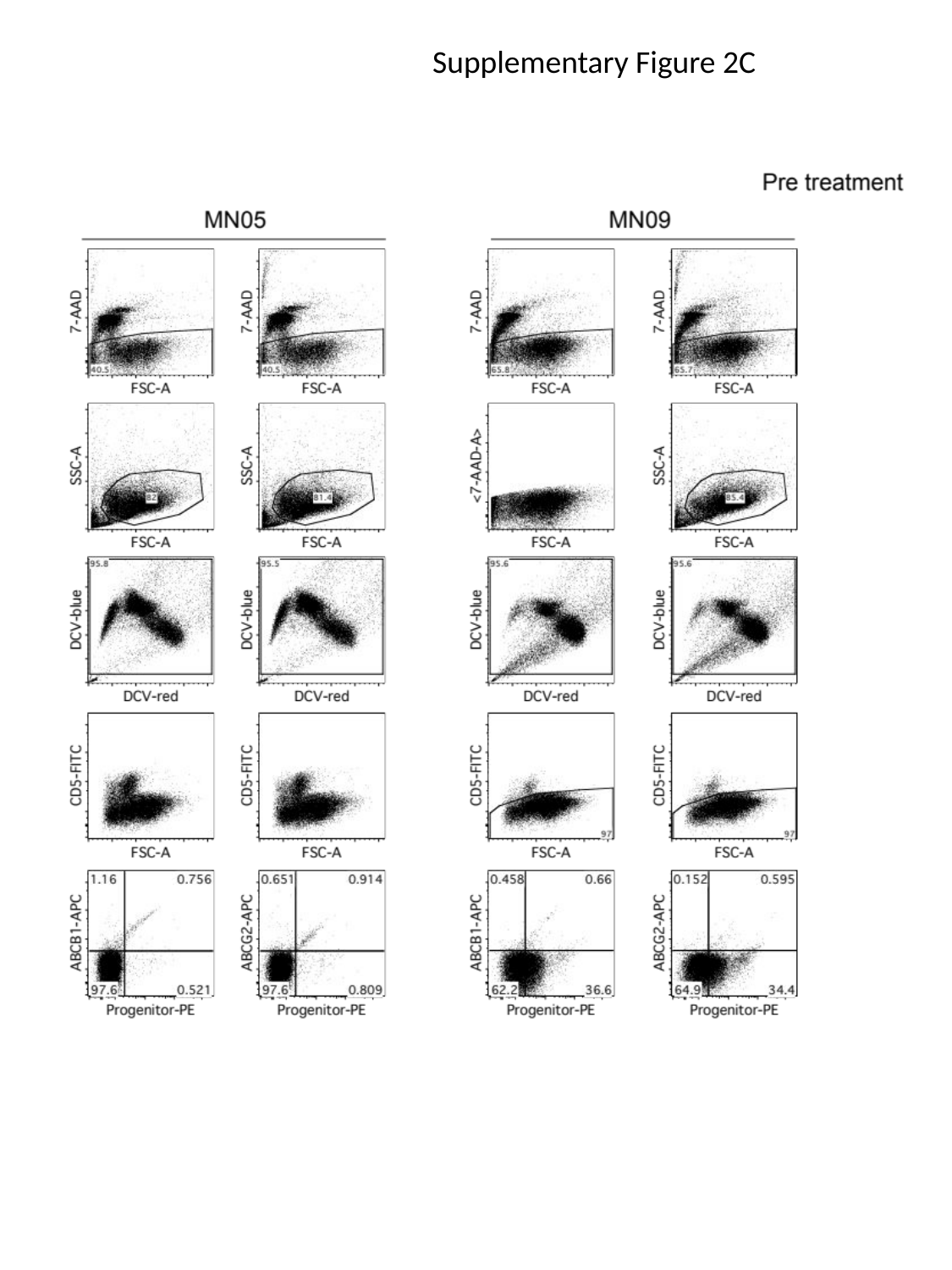

Supplementary Figure 2C

## Slide 8
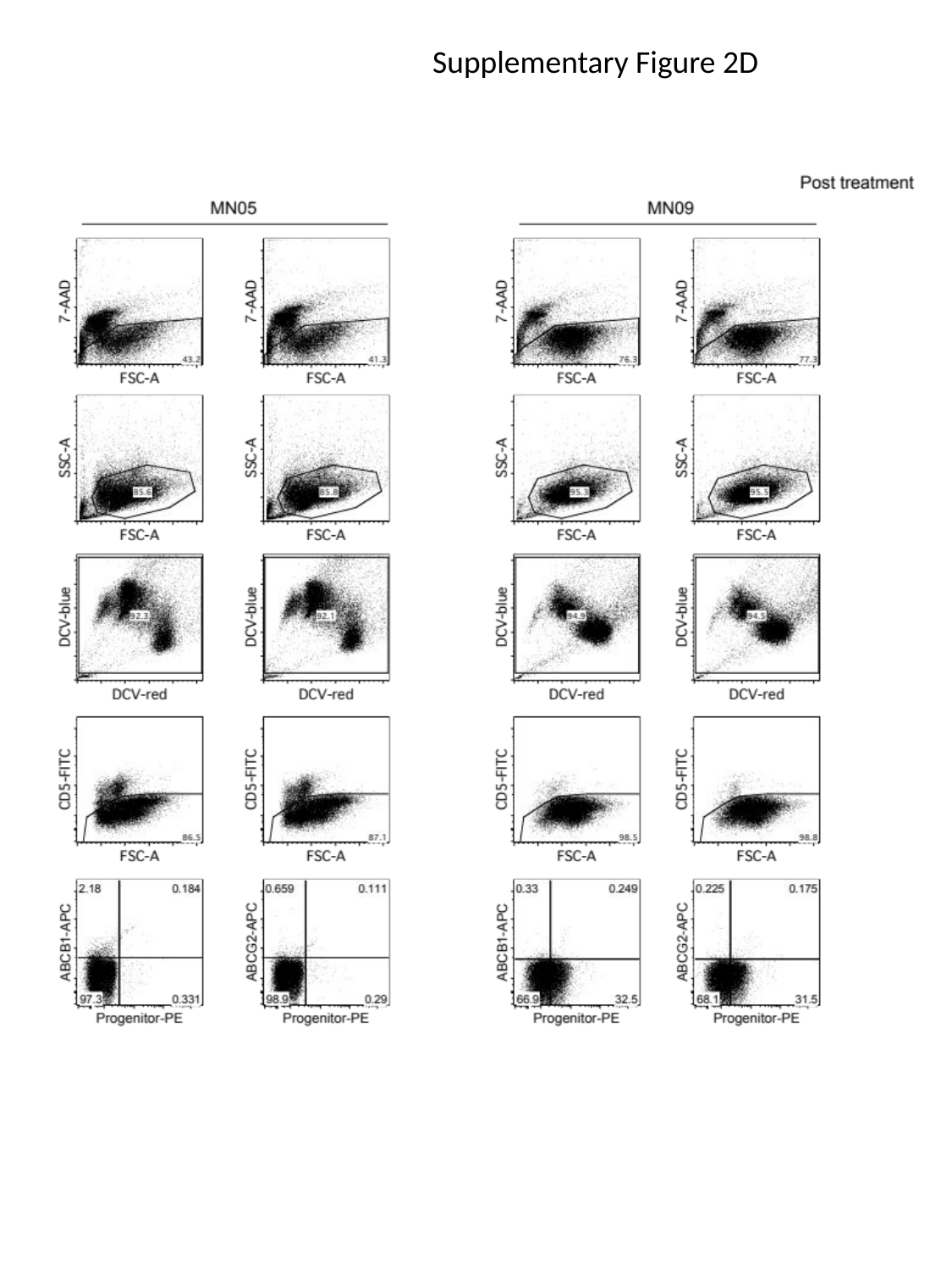

Supplementary Figure 2D

## Slide 9
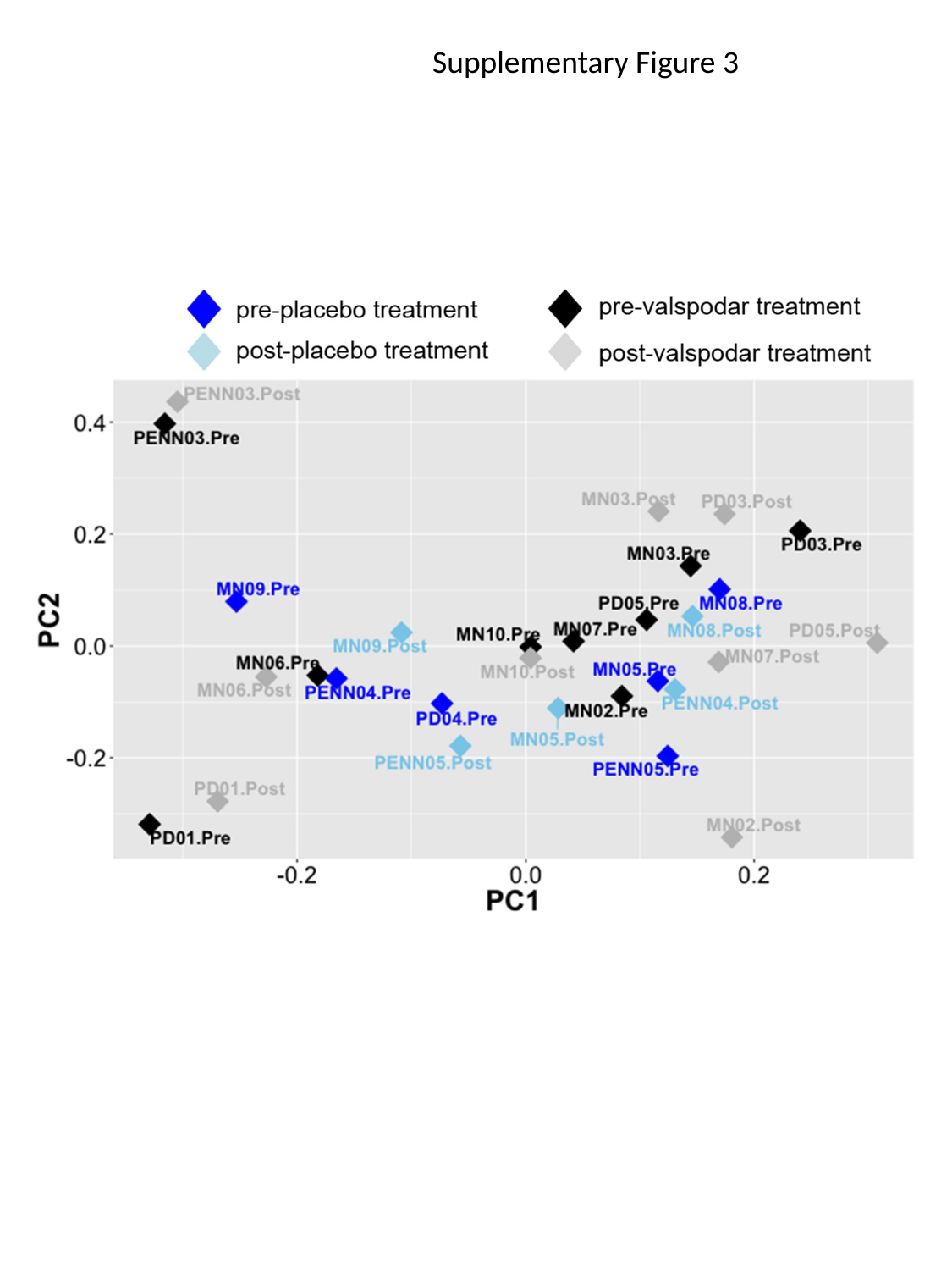

Supplementary Figure 3
