# Supplementary material for: A double blinded, placebo-controlled pilot study to examine reduction of CD34 +/CD117 +/CD133 + lymphoma progenitor cells and duration of remission induced by neoadjuvant valspodar in dogs with large B-cell lymphoma
Source: F1000Res. 2017 Apr 18;4:42. Originally published 2015 Feb 11. [Version 3] doi: 10.12688/f1000research.6055.3 (PMC5357040; doi:10.12688/f1000research.6055.3)
Supplement: Peer review history

# Peer review: Referee response for version 2

**DOI:** 10.5256/f1000research.11536.r20948

**Published:** 2017-03-14

**Authors:** Douglas H. Thamm

**Affiliations:** 1 Flint Animal Cancer Center, Colorado State University, Fort Collins, CO, USA

I have read the new version of the manuscript, and I believe my concerns have been adequately addressed.

I have read this submission. I believe that I have an appropriate level of expertise to confirm that it is of an acceptable scientific standard.

# Peer review: Referee response for version 2

**DOI:** 10.5256/f1000research.11536.r20743

**Published:** 2017-03-07

**Authors:** Michael S. Kent

**Affiliations:** 1 Department of Surgical and Radiological Sciences, School of Veterinary Medicine, University of California, Davis, Davis, CA, USA

I have reviewed the author responses and they have addressed my concerns adequately.

I have read this submission. I believe that I have an appropriate level of expertise to confirm that it is of an acceptable scientific standard.

# Peer review: Referee response for version 1

**DOI:** 10.5256/f1000research.6482.r8911

**Published:** 2015-06-05

**Authors:** Michael S. Kent

**Affiliations:** 1 Department of Surgical and Radiological Sciences, School of Veterinary Medicine, University of California, Davis, Davis, CA, USA

The manuscript entitled “A double blinded, placebo-controlled pilot study to examine reduction of CD34+/CD117+/CD133+ lymphoma progenitor cells and duration of remission induced by neoadjuvant valspodar in dogs with large B-cell lymphoma” presents a prospective study evaluating the effects of valspodar in a single agent setting for effects on LPCs  and as a secondary endpoint evaluates outcomes in dogs treated with valspodar and doxorubicin for B-cell lymphoma.

The manuscript is well written and describes the study design and materials and methods well.

My main concern with the manuscript is that the main outcome studied was a change in LPCs before and 4 days after starting valspodar. Given the mechanism of action of the drug in inhibiting the ABC transporter and not cytotoxcity of these cells specifically I am not surprised that the authors did not see a drop in their numbers.  It is unfortunate that there were only a few samples with enough material to test if valspodar could inhibit the ABC transporters *in vivo *as this would have been a very useful component of the study. In my opinion these points should be in the discussion. 

While underpowered I think the length of remission and overall survival data are important as they show that there is not likely to be differences in these groups even in a larger study.

I have read this submission. I believe that I have an appropriate level of expertise to confirm that it is of an acceptable scientific standard, however I have significant reservations, as outlined above.

# Response

**Published:** 2017-01-15

**Authors:** Jaime Modiano

**Affiliations:** Vet Clin Sci and Cancer Center, University of Minnesota, USA

**Comment: **The manuscript entitled “A double blinded, placebo-controlled pilot study to examine reduction of CD34+/CD117+/CD133+ lymphoma progenitor cells and duration of remission induced by neoadjuvant valspodar in dogs with large B-cell lymphoma” presents a prospective study evaluating the effects of valspodar in a single agent setting for effects on LPCs and as a secondary endpoint evaluates outcomes in dogs treated with valspodar and doxorubicin for B-cell lymphoma.

 The manuscript is well written and describes the study design and materials and methods well.

**Response: **We appreciate the reviewer’s positive comments

**Comment: **My main concern with the manuscript is that the main outcome studied was a change in LPCs before and 4 days after starting valspodar. Given the mechanism of action of the drug in inhibiting the ABC transporter and not cytotoxcity of these cells specifically I am not surprised that the authors did not see a drop in their numbers.  It is unfortunate that there were only a few samples with enough material to test if valspodar could inhibit the ABC transporters in vivo as this would have been a very useful component of the study. In my opinion these points should be in the discussion.

**Response: **This comment (as well as a comment from Reviewer #2) made it clear to us that we failed to explain clearly why we hypothesized that LPCs would be depleted in dogs treated with neoadjuvant valspodar followed by doxorubicin. This failure was partly due to circumstances explained in our cover letter to the editors, and our efforts to correct it were the main reason it took so long for us to submit a response. In fact, we agree with the reviewer that valspodar is not cytotoxic and should not cause a reduction in LPSc by itself. Rather, our prediction was that by reducing the activity of ATP-binding cassette transporter (ABC) proteins, including ABCB1 (also known as P-glycoprotein), valspodar would sensitize LPCs to the cytotoxic effects of doxorubicin.  We expected doxorubicin treatment to induce rapid clinical remission with consequent lymphoid necrosis and lymphodepletion of malignant nodes. Thus, we felt that quantification of LPCs from this nodal environment would be challenging. Instead, we decided to measure depletion of LPCs in peripheral blood as a surrogate measure of valspodar-induced sensitization to doxorubicin. As noted in the materials and methods section, blood was collected from each dog on day 11 of the study, 7 days after administration of doxorubicin.  We believe this was a reasonable time point for sampling blood to investigate whether circulating LPCs were sensitized by the neoadjuvant valsopodar and depleted by doxorubicin treatment in treated dogs as compared to controls.

**Comment: **While underpowered I think the length of remission and overall survival data are important as they show that there is not likely to be differences in these groups even in a larger study.

**Response: **We appreciate the reviewer’s comment. We agree, and that is the reason why we included the data in the original manuscript and kept this section unchanged.

# Peer review: Referee response for version 1

**DOI:** 10.5256/f1000research.6482.r8454

**Published:** 2015-05-01

**Authors:** Douglas H. Thamm

**Affiliations:** 1 Flint Animal Cancer Center, Colorado State University, Fort Collins, CO, USA

This is a very well written and articulate manuscript exploring the effects of valspodar or placebo on putative tumor-initiating cell number and clinical outcome in dogs treated with doxorubicin. There is very encouraging preliminary data identifying these putative tumor-initiating cells and documenting their high expression of efflux pumps such as P-glycoprotein. Unfortunately there are 2 major issues with conception/design of the study, which are a cause for concern and need additional justification/clarification if the manuscript was to be considered for indexation.

1. As written, it appears that changes in lymphoid progenitor cell percentages in patient dogs were assessed before and after valspodar treatment, but before any chemotherapy was given (“Trial design” section and Table 2). The role of P-GP in mediating chemotherapy sensitivity is through facilitating the cellular efflux of certain cytotoxic drugs. In the absence of these cytotoxic drugs, P-GP inhibitors would be expected to have no independent cytotoxic effect on any cell population. Thus, the absence of a change in LPC percentage (or gene expression) is intuitive based on the mechanism of the drug and the study as-designed. If LPC number was assessed following doxorubicin (+/- valspodar) treatment, a difference MIGHT have been observed. If I am somehow mistaken about the study design, then it needs substantial clarification.
2. Extensive clinical evaluation of valspodar/chemotherapy combinations in humans has failed to demonstrate an improvement in outcome, even in very large randomized phase-3 trials. For example, CALGB 19808 randomized 302 patients with AML to chemotherapy or chemotherapy plus valspodar – response rates, DFS and OS were no different and were actually numerically shorter in the valspodar arm ( Kolitz *et al*, 2010). A second study randomized 762 patients with ovarian cancer to carboplatin/paclitaxel +/- valspodar – no difference in outcome was observed ( Lhommé *et al*., 2008). A third randomized study, ECOG E1A95, evaluated VAD +/- valspodar in 94 patients with refractory myeloma. No difference in outcome was observed ( Friedenberg *et al*, 2006). Although an alternate mechanism for drug efficacy is invoked in this study, it seems counterintuitive to think that differences in outcome (although a secondary measure) would be observed in a study of 20 dogs with lymphoma. None of the above-mentioned studies were discussed or cited in the Introduction or Discussion.

I have read this submission. I believe that I have an appropriate level of expertise to state that I do not consider it to be of an acceptable scientific standard, for reasons outlined above.

# Response

**Published:** 2017-01-15

**Authors:** Jaime Modiano

**Affiliations:** Vet Clin Sci and Cancer Center, University of Minnesota, USA

**Comment: **This is a very well written and articulate manuscript exploring the effects of valspodar or placebo on putative tumor-initiating cell number and clinical outcome in dogs treated with doxorubicin. There is very encouraging preliminary data identifying these putative tumor-initiating cells and documenting their high expression of efflux pumps such as P-glycoprotein.

**Response: **We appreciate the reviewer’s positive comments

 Unfortunately there are 2 major issues with conception/design of the study, which are a cause for concern and need additional justification/clarification if the manuscript was to be considered for indexation.

**Comment: **As written, it appears that changes in lymphoid progenitor cell percentages in patient dogs were assessed before and after valspodar treatment, but before any chemotherapy was given (“Trial design” section and Table 2). The role of P-GP in mediating chemotherapy sensitivity is through facilitating the cellular efflux of certain cytotoxic drugs. In the absence of these cytotoxic drugs, P-GP inhibitors would be expected to have no independent cytotoxic effect on any cell population. Thus, the absence of a change in LPC percentage (or gene expression) is intuitive based on the mechanism of the drug and the study as-designed. If LPC number was assessed following doxorubicin (+/- valspodar) treatment, a difference MIGHT have been observed. If I am somehow mistaken about the study design, then it needs substantial clarification.

**Response: **We agree with the reviewer, and refer to our response to comments from reviewer 1 and our cover letter to the editors for a detailed explanation. Moreover, we re-analyzed the RNA sequencing data, and corrected a technical error in our previous statement, although it does not change the interpretation of the data. In fact, there were observed genes whose expression was significantly different in pre-treatment and post-treatment groups. However, we did not find consistent, genome-wide changes in gene expression that could be attributed to drug treatment (valspodar vs. placebo) or to time (Day 0 vs. Day 4). A more precise description of the methods used, the results, and the interpretation are included in the revised manuscript.

**Comment: **Extensive clinical evaluation of valspodar/chemotherapy combinations in humans has failed to demonstrate an improvement in outcome, even in very large randomized phase-3 trials. For example, CALGB 19808 randomized 302 patients with AML to chemotherapy or chemotherapy plus valspodar – response rates, DFS and OS were no different and were actually numerically shorter in the valspodar arm (Kolitz et al, 2010). A second study randomized 762 patients with ovarian cancer to carboplatin/paclitaxel +/- valspodar – no difference in outcome was observed (Lhommé et al., 2008). A third randomized study, ECOG E1A95, evaluated VAD +/- valspodar in 94 patients with refractory myeloma. No difference in outcome was observed (Friedenberg et al, 2006). Although an alternate mechanism for drug efficacy is invoked in this study, it seems counterintuitive to think that differences in outcome (although a secondary measure) would be observed in a study of 20 dogs with lymphoma. None of the above-mentioned studies were discussed or cited in the Introduction or Discussion.

**Response: **The reviewer is correct that valspodar had been evaluated previously in three large phase-3 clinical trials with no evidence of improved response rates. Our intent was to determine if inhibition of ABC transporters, and ABCB1 in particular, would sensitize LPCs to the cytotoxic effects of doxorubicin, presumably by increasing the retention time of the drug in the cells. Our study was not powered to detect differences in duration of remission or overall survival, but differences in LPCs might have supported a revised study design for new trials in cancers of dogs or humans that are presumed to be driven by tumor-initiating or tumor-propagating cells with elevated ABC transporter activity. We appreciate the reviewer’s point, that clarifying the previous use of valspodar in cancer patients and how this study was meant to build on the negative data, and specifically how the design differed from those studies, is valuable for context. We have added this information to the Introduction and Discussion sections.
